# Data driven selection of consensus docking pipelines for structure based hit identification

**DOI:** 10.1038/s44386-026-00063-4

**Published:** 2026-09-02

**Authors:** Hamza Agha, Youssef Ibrahim, Michael Backenköhler, Antoine Lacour, Mostafa M. Hamed, Anna K. H. Hirsch, Andrea Volkamer

**Affiliations:** 1 Data-Driven Drug Design, Center for Bioinformatics, Saarland Informatics Campus, Saarland University, Saarbrücken, Saarland Germany; 2 Helmholtz Institute for Pharmaceutical Research Saarland (HIPS), Saarbrücken, Saarland Germany; 3 PharmaScienceHub (PSH), Saarbrücken, Saarland Germany; 4 Department of Pharmacy, Saarland University, Saarbrücken, Saarland Germany; 5 Present Address: Helmholtz Institute for Pharmaceutical Research Saarland (HIPS), Saarbrücken, Saarland Germany

**Keywords:** Computational biology and bioinformatics, Drug discovery

## Abstract

Structure-based virtual screening (SBVS) is a cornerstone of computer-aided drug design, yet its success depends on selecting a combination of docking tools, scoring function (SF), and ranking strategies. MolDockLab addresses this challenge with an automated, data-driven framework that optimizes SBVS workflows for a protein target, balancing predictive performance and computational efficiency. It systematically explores combinations of five docking engines, 15 SF, and three consensus ranking strategies using a calibration set of ≈ 200 compounds with known bioactivity, and applies the best-correlating workflow to the larger screening library. Final hit selection from the top 1% integrates protein-ligand interaction profiler (PLIP)-derived interaction fingerprints, structural-diversity assessment, and expert visual inspection. In a retrospective evaluation on the epidermal growth factor receptor (EGFR), the chosen pipeline achieved a Spearman correlation of 0.36 and an enrichment factor (EF) at 10% of 1.57, consistent with calibration. Prospectively, for the energy coupling factor transporters (ECF-T)-a challenging transmembrane target with a cryptic binding site and no co-crystallized ligand-the pipeline reached a correlation of 0.45 and enrichment of 3.13. Post-processing enabled in vitro confirmation of two chemically novel inhibitors rivaling the most potent ECF-T inhibitors reported to date.

## Introduction

Drug discovery is inherently resource- and time-intensive, often requiring up to 14 years and approximately 879.3 million USD per approved drug^1,2^. A critical early milestone is the identification of promising chemical “hits”. This task is typically accomplished through high-throughput screening (HTS) of large compound libraries. HTS is implemented in two main formats: phenotypic assays (forward pharmacology), which detect compound-induced cellular or organismal responses without prior target knowledge, and target-based assays (reverse pharmacology), which quantify the modulation of a predefined biomolecular target hypothesized to yield therapeutic benefit^2–5^. Even with these established methodologies, substantial scope remains to reduce both time and cost in the early discovery and preclinical phases^6^.

Rational drug design, also known as reverse pharmacology, employs a structure-based approach that begins with the identification of molecular targets and proceeds to identify high-affinity compounds^3^. Using this approach, a viable drug candidate can be delivered in roughly two years, compared with the approximate five years usually required by forward pharmacology^3^. To expedite the hit-identification process still further, computational methods have gained significant attention. Among computational strategies, Structure-based virtual screening (SBVS) has emerged as a pivotal technique in modern rational drug design. By leveraging three-dimensional (3D) protein structures, SBVS systematically evaluates and ranks prospective ligands from vast chemical libraries, substantially reducing the experimental workload^2,7^. A central element of SBVS is molecular docking, which predicts the binding pose of each ligand within a protein’s active site and estimates the interaction strength through a scoring function (SF)^2,8^.

A wide range of molecular docking software and SFs are available, each leveraging distinct approaches. Broadly, molecular docking approaches can be categorized, as summarized in Table 1, as follows: (i) *Stochastic methods* heuristically adjust a ligand’s degrees of freedom, enabling rapid exploration of diverse poses, though potentially overlooking optimal conformations due to limited exploration. Algorithms based on swarm optimization, such as ant colony optimization employed in protein-ligand ANT System (PLANTS), leverage collective behavior simulations to efficiently identify energy-minimal paths by exploiting previously successful poses^9^. Genetic algorithms, utilized by docking tools including AutoDock^10^, rDOCK^11^, GOLD^12^, and iGEMDOCK^13^, apply mechanisms of mutation and crossover to iteratively refine ligand conformations toward optimal fitness scores. Similarly, tabu search strategies, as seen in PSI-DOCK^14^, enhance pose discovery by avoiding re-evaluation of previously explored regions. The Metropolis Monte Carlo approach, incorporated in AutoDock Vina^15^, ICM^16^, MCDOCK^17^, and QXP^18^, randomly alters ligand conformations to navigate energy landscapes, occasionally accepting less favorable states to escape local energy minima^2^. (ii) *Systematic exploration methods* methodically assess configurations within a predefined parameter space^2^. Glide’s docking protocol^19^ employs exhaustive search strategies, systematically analyzing all rotatable bonds while imposing constraints to limit the search space. Incremental construction methods, as employed by FlexX^20^ and Hammerhead^21^, dock an initial core fragment first, subsequently adding the remaining fragments incrementally to form a complete ligand. (iii) *Deep learning models* have recently emerged as powerful tools to classical approaches for protein-ligand docking and binding-pose prediction. In 2022, early methods such as EquiBind^22^ and TANKBind^23^ predicted both binding sites and 3D ligand conformations, a process known as blind docking, although these regression-based approaches showed limited improvement in binding-pose accuracy^23^. In 2023, DiffDock introduced an innovative diffusion generative model that learns ligand orientation distributions on SE(3), significantly improving pose prediction accuracy and demonstrating superior binding site identification capabilities^24^. The same year, KarmaDock^25^ introduced a three-stage architecture combining E(n)-equivariant graph neural networks with self-attention and post-processing correction to accelerate large-library docking while improving pose quality and binding affinity estimation. In 2024, DiffDock-L^24^ addressed generalizability issues inherent in its predecessor, while SurfDock^26^ further advanced diffusion-based docking by integrating protein sequence, 3D structural graphs, and surface-level features into an equivariant architecture operating on a non-Euclidean manifold, achieving state-of-the-art docking success rates with enhanced physical plausibility and generalizability to unseen proteins.

**Table 1:** Summary of molecular docking tools classified by search strategy

Class	Algorithm	Tool(s)
Stochastic	Ant Colony Optimization	PLANTS^9^
	Genetic Algorithm (GA)	AutoDock^10^, rDOCK^11^, GOLD^12^, iGEMDOCK^13^
	Tabu Search + GA	PSI-DOCK^14^
	Monte Carlo (Metropolis)	AutoDock Vina^15^, ICM^16^, MCDOCK^17^, QXP^18^
Systematic	Exhaustive Search	Glide^19^
	Incremental Construction	FlexX^20^, Hammerhead^21^
Deep Learning	Geometric / Regression	EquiBind^22^, TANKBind^23^
	Equivariant GNN	KarmaDock^25^
	Diffusion Generative	DiffDock, DiffDock-L^24^, SurfDock^26^
	Co-Folding	AlphaFold 3^27^, Boltz-1^28^, Boltz-2/2x^29^

Beyond docking-specific models, co-folding approaches that jointly predict protein and ligand 3D structures have significantly advanced the field. AlphaFold 3^27^ demonstrated accurate prediction of biomolecular complex structures across diverse modalities. Boltz-1^28^ offered the first fully open-source co-folding model approaching AlphaFold 3 accuracy, with novel inference-time steering potentials to correct non-physical predictions. Most recently, Boltz-2/2x^29^ extended these capabilities by jointly modeling complex structures and binding affinity, approaching the accuracy of physics-based free-energy perturbation methods while being over 1000 times faster. Its variant Boltz-2x employs physicality steering potentials at inference time to further reduce steric clashes and improve structural plausibility.

Despite these rapid advances, critical limitations remain. Emerging evidence suggests that deep learning-based docking and co-folding models are prone to overfitting to training-set poses, reproducing learned binding patterns instead of genuinely modeling the physics of protein-ligand interactions^30–32^. The PoseBusters benchmark^32^ revealed that deep-learning methods frequently generate physically implausible poses and fail to generalize to protein-ligand complexes absent from their training data, with classical docking tools outperforming all tested deep-learning methods on out-of-distribution targets. Masters et al.^31^ further demonstrated that co-folding models, including AlphaFold 3 and Boltz-1, continue to predict nearly identical ligand poses even after critical binding-site residues are mutated to abolish favorable interactions, indicating reliance on memorized structural patterns rather than true molecular-interaction principles. A recent large-scale evaluation of Boltz-2 across approximately 38,000 compounds for two therapeutically relevant targets revealed that the model exhibits only weak to moderate correlation with physics-based binding free energies and yields no significant correlation for its top-ranked candidates^33^.

SFs are essential in SBVS for ranking predicted poses and evaluating the binding energies of diverse ligand conformations. However, SF fall short in accurately capturing the complex physical dynamics of protein-ligand interactions. Instead, SFs typically serve as an approximation of binding energies, aiming to balance computational efficiency with predictive accuracy^34^. SFs can be categorized into four main types: physics, empirical, knowledge, and machine learning-based.

*Physics-based SF*, derived from classical or quantum force fields, calculate interactions including electrostatic and van der Waals forces, often enhanced by accounting for entropy and solvation effects through models such as COMBINE^35^, MedusaScore^36^, GoldScore^37^ and SQM 2.20^38^. *Empirical SF*, including CHEMPLP^39^, SMINA’s SF^40^, Vinardo scores^41^, HYDE^42,43^, LinF9^44^ and AD4^10^, use weighted energy terms representing various interaction forces, calibrated against known binding data. *Knowledge-based SF* leverage statistical potentials from extensive datasets of protein-ligand interactions, exemplified by SMoG^45^ and potential of mean force SF^46^, which rely on the frequency of atomic pair interactions compared to a reference state to predict energetically favorable conformations. *Machine learning-based SF* employ algorithms to learn protein-ligand complex interaction patterns and predict binding affinities. RFScore applies random forest (RF)^47^, while GNINA integrates convolutional neural networks (CNN)^48^. SCORCH combines ensemble machine-learning techniques for improved classification of actives^49^. Furthermore, RTMScore employs a novel SF based on residue-atom distance likelihood potentials and graph-transformer architectures^50^.

Benchmarking studies have repeatedly demonstrated that docking performance is highly system-dependent, with no single SF or search algorithm emerging as universally superior across all target classes and ligand collections^51,52^. Additionally, given that both docking algorithms and SF vary widely in their accuracy and reliability, there has been a shift towards the implementation of consensus docking strategies. This approach involves the concurrent run of multiple docking engines to improve the robustness and accuracy of the virtual screening^2,53^. In a complementary strategy known as consensus scoring, the results of multiple SFs are combined so that compounds repeatedly ranked highly by different metrics are prioritized^54,55^. Consensus schemes fall into two broad classes: score-based methods (e.g., rank-by-number, Z-score, and the augmented Z-score that averages the best two of three scores) and rank-based methods (e.g., rank-by-rank (RbR), rank-by-vote (RbV), reciprocal rank, Pareto ranking, and exponential consensus ranking (ECR))^54–56^. By aggregating outputs from various docking engines and distinct categories of SF, consensus approaches reduce individual biases and systematically improve the predictive accuracy of SBVS^2,53–55,57^. Moreover, because each docking run typically produces multiple candidate poses per ligand, a pose selection step is required to reduce these to a single representative binding mode before scoring and ranking^58^. Common strategies include retaining the top-scored pose, clustering geometrically similar poses and selecting a cluster representative^58,59^, or applying interaction-based filters^60^.

DockM8^57^ has emerged as an innovative consensus SBVS tool that integrates a comprehensive suite of five distinct docking algorithms, 16 SF, 16 pose selection strategies for narrowing down the multitude of generated poses, and 6 methods for consensus ranking. By employing both consensus-docking and consensus-scoring techniques, it successfully identifies compounds that consistently achieve high-ranking scores. By selecting the most effective SBVS workflow for each target (named DockM8-max), it outperformed individual docking tools and SFs benchmarking DUD-E^61^, DEKOIS 2.0^62^, and LIT-PCBA^63^ datasets, achieving higher hit rates and enrichment factor (EF). Furthermore, when a set of a few known actives is supplied, DockM8 can automatically generate matched decoys using DeepCoy^64^, allowing it to identify the pipeline that yields the highest EF. However, DockM8 selects pipelines by maximizing binary screening power (EF, BEDROC, AUC-ROC) on actives-vs-decoys/inactives sets and outputs a ranked compound list. To address this challenge, we developed MolDockLab, which is designed for a complementary scenario: when a small set of compounds with measured continuous potency values is available against the target, the pipeline is selected by maximizing rank correlation with those potencies, with explicit weighting of computational cost, and the workflow continues past ranking into automated protein-ligand interaction profiler (PLIP)-based interaction filtering and K-medoids diversity clustering before final expert inspection. When activity data are heterogeneous, mixing pIC_50_values with single-concentration inhibition, a Hodge-ranking step can be applied upstream to consolidate them into one ranking before they enter the MolDockLab workflow.

We developed MolDockLab to determine the optimal SBVS workflow from all possible combinations among five docking programs, 15 SFs and energy terms, and three consensus ranking methods, producing a maximum of 3,047,361 pipelines. To avoid further combinatorial explosion, MolDockLab retains the top-scored pose from the selected pipeline for each compound, and pose selection is not treated as an additional tunable factor. Each candidate workflow is benchmarked on a small calibration subset comprising compounds with known experimental activity data against the target of interest. The predictive accuracy of each workflow is then weighed against wall-clock runtime to balance performance with computational cost. Once the top-ranked pipeline is chosen, MolDockLab automatically applies it to the full screening set and carries out post-processing for hit selection, thereby streamlining the entire path from initial assessment to hit identification.

As a retrospective benchmark, we selected the epidermal growth factor receptor (EGFR, also known as ErbB1/HER1), a member of the ErbB receptor-tyrosine-kinase family whose extensive structural and biochemical annotation makes it a standard test bed for SBVS methods^65^. Prospectively, we targeted energy coupling factor transporters (ECF-T), which are bacterial micronutrient transporters that have emerged as promising antibacterial targets^66,67^. In the ECF-T study, the calibration data was obtained from in-house assays conducted at the HIPS. Applying MolDockLab to both EGFR (validation) and ECF-T (hit discovery) enabled us to demonstrate the workflow’s effectiveness for established SBVS benchmarks as well as for forward-looking lead-identification campaigns. In particular, the ECF-T system poses exceptional challenges for structure-based approaches, as it is a multi-subunit transmembrane complex with a dynamic, membrane-buried cryptic pocket, no co-crystallized ligand, and no previously reported inhibitors from virtual screening, making it a stringent real-world test of any computational hit-identification strategy.

## Results

**Fig. 1:**
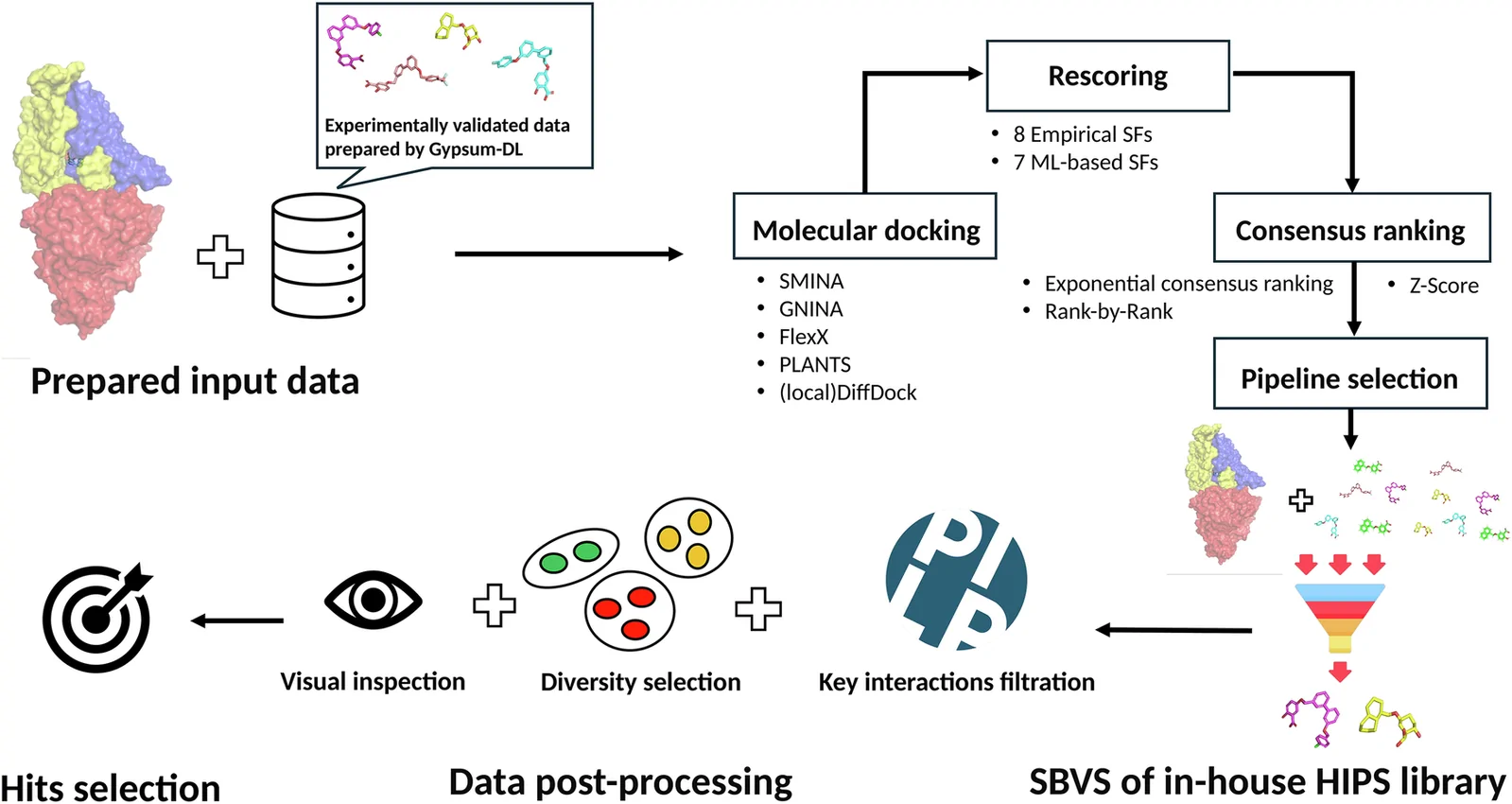
Overview of the MolDockLab data-driven workflow for structure-based virtual screening and hit identification. The workflow takes a prepared protein structure and a calibration set of compounds with known bioactivity as input. It deploys five docking tools and 15 scoring functions (SFs), and aggregates their outputs with three consensus-ranking methods, to select an efficient pipeline that balances predictive performance and computational cost. The selected pipeline is then applied to the full screening library. Top-ranked compounds are refined through protein--ligand interaction-fingerprint filtering, structural-diversity selection by clustering, and expert visual inspection to yield the final hit list. SBVS, structure-based virtual screening.

To evaluate MolDockLab, we applied the full workflow (Fig. 1) to two targets of differing data availability. For EGFR, a calibration set of 201 compounds with known pIC_50_values was used to benchmark all candidate pipelines, totaling 95,139 combinations of docking tools, SFs, and consensus-ranking methods. Then, we deployed the selected pipeline on the screening set of 6473 compounds. For ECF-T, a calibration set of 212 compounds (activity harmonized via Hodge ranking) guided the evaluation of 190,371 pipeline variants, after which the optimal pipeline was applied to screen the 6505 compound in-house library. In both cases, five docking engines were involved, each generating up to ten poses per ligand. All poses were re-scored with the retained panel of non-redundant SF, and three consensus-ranking strategies were compared. Pipeline selection balanced Spearman correlation, EF_10%_, and runtime cost. For the ECF-T target, the top-ranked compounds additionally underwent PLIP-based interaction filtering, K-medoids diversity clustering, and expert visual inspection, culminating in 17 candidates submitted for in vitro testing.

### Benchmarking of individual workflow components

To systematically characterize the behavior of individual workflow components, we first examined the coverage of successful docking runs per target and pipeline, followed by an assessment of pose quality and physical plausibility. We then analyzed scoring-function behavior, including redundancy and variability, before evaluating how different consensus-ranking strategies influence ranking accuracy and computational cost.

With regard to docking-pose coverage, for the EGFR calibration set of the 201 ligands, a total of 9755 poses were generated (Table 2). SMINA and GNINA successfully processed all compounds, producing ten poses per compound without any rejections. FlexX docked 199 compounds, resulting in 1929 poses. PLANTS processed 193 compounds (1929 poses), corresponding to a 4% shortfall, while DiffDock docked 188 compounds (1877 poses), reflecting a 7% reduction in coverage. For the ECF-T target, localDiffDock, SMINA, and GNINA again achieved full processing, each generating ten poses per compound. FlexX also docked all compounds, producing 2101 poses. In contrast, PLANTS docked 210 compounds and generated 2090 poses, indicating a 1.5% reduction in coverage. Overall, a majority of compounds could be docked with a sufficient number of pose suggestions, enabling a comprehensive evaluation of structural quality.

**Table 2:** Number of successfully docked compounds (with success rate) and total generated poses per docking tool for the epidermal growth factor receptor (EGFR) and energy-coupling factor transporter (ECF-T) targets

	EGFR (201 compounds)		ECF-T (212 compounds)	
Docking tool	Compounds (Success %)	Poses	Compounds (Success %)	Poses
SMINA	201 (100%)	2010	212 (100%)	2120
GNINA	201 (100%)	2010	212 (100%)	2120
FlexX	199 (99%)	1929	212 (100%)	2101
PLANTS	193 (96%)	1929	210 (99%)	2090
(local)DiffDock	188 (93%)	1877	212 (100%)	2120
**Total**	—	9755	—	10551

Each tool was configured to produce up to ten poses per compound.

All generated poses were subsequently assessed with PoseBusters for chemical validity, intramolecular strain, and intermolecular plausibility; the complete per-tool, per-target breakdown is provided in Supplementary Fig. 6. Chemical validity was essentially universal across tools and targets. The tools differed mainly in how well they controlled intramolecular strain and, more decisively, in the quality of the resulting intermolecular geometries. FlexX produced the fewest intramolecular issues overall, while SMINA and GNINA showed slightly more high-energy conformers but achieved near-perfect adherence to intermolecular criteria on both targets. PLANTS performed robustly but with moderate violations on both fronts. DiffDock (and localDiffDock on ECF-T) showed substantial shortcomings in intermolecular validation, with the vast majority of poses violating minimum protein–ligand distance and overlapping with the receptor volume. These complementary strengths and weaknesses indicate that no single tool is universally optimal, and motivate combining outputs from multiple methods to obtain a more reliable docking ensemble in the subsequent analysis.

Next, all generated poses were evaluated with the full set of SF, and their predicted scores were normalized. The resulting score-range distributions are reported in Supplementary Fig. 3 in the Supplementary Information. To avoid redundancy and reduce noise in the downstream analysis, SF exhibiting high pairwise correlation (*r* ≥ 0.9) and low correlation with the experimental values were excluded, as detailed in Supplementary Figs. 4 and 5. For the EGFR target, following SFs were removed from further consideration: RFscore V1, LinF9, AD4, Vinardo, and RFscore V3. For the ECF-T target, RFscore V2, SMINA affinity, LinF9, and Vinardo were excluded. The remaining SFs were carried forward for subsequent affinity prediction and ranking analysis.

Following this SF reduction, the retained pipelines were used to evaluate how different ranking strategies affect overall pipeline performance. Each candidate pipeline is defined by a unique combination of one or more docking tools, one or more SFs, and a single consensus-ranking strategy. After removing redundant SFs (see “Scoring function selection”), 10 and 11 non-redundant SFs were retained for EGFR and ECF-T, respectively. The combinatorial expansion over all subsets of docking tools and retained SFs yields 31,713 and 63,457 docking tool-SF combinations for EGFR and ECF-T, respectively, producing 95,139 and 190,371 scored pipeline results in total when crossed with the three ranking methods.

**Fig. 2:**
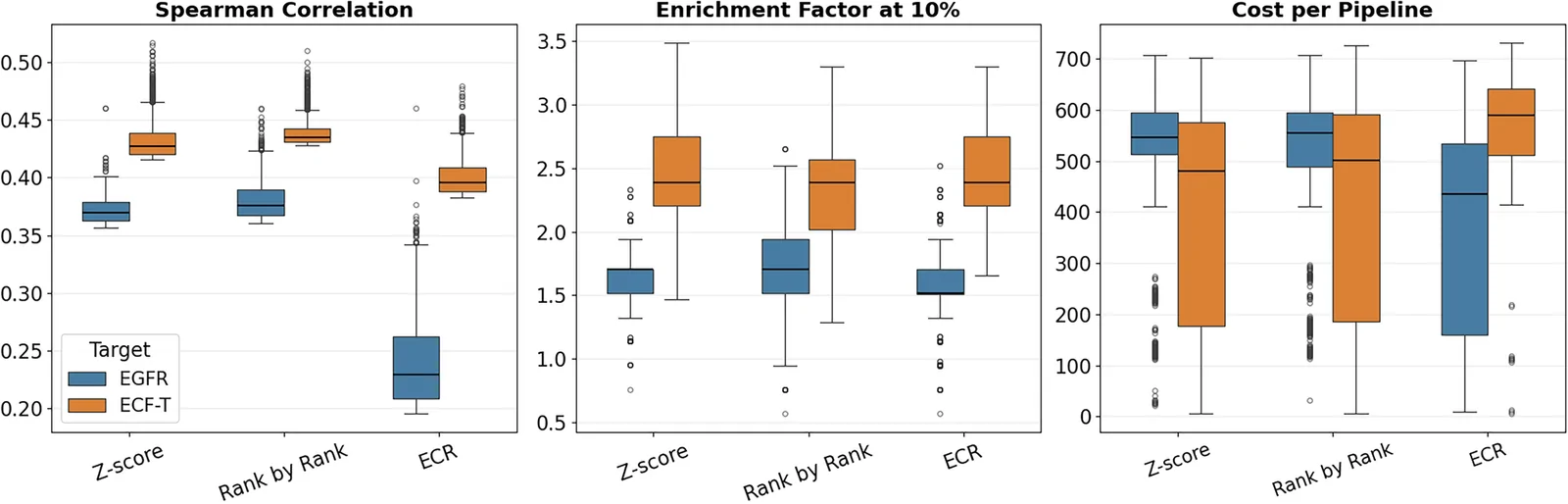
Benchmark of three consensus-ranking strategies across the top 1% of virtual-screening pipelines for both targets. Performance of the Z-Score, rank-by-rank (RbR), and exponential consensus ranking (ECR) strategies, evaluated on the top 1% of pipelines for the epidermal growth factor receptor (EGFR, blue boxes) and the energy-coupling factor transporter (ECF-T, orange boxes). The left panel shows the Spearman correlation coefficient between predicted and experimental rankings; the center panel shows the enrichment factor at 10%; the right panel shows the computational cost per compound. In each box-and-whisker plot, the box spans the interquartile range, the horizontal line marks the median, the whiskers extend to 1.5 times the interquartile range, and individual points beyond the whiskers are outliers.

We selected the top 1% of the pipelines based on Spearman correlation (*ρ*) relative to experimental affinity values. Among the three tested consensus-ranking strategies (Z-Score, RbR, and ECR), both Z-Score and RbR achieved the highest median correlations (EGFR: *ρ* ≈ 0.36to0.37; ECF-T: *ρ* ≈ 0.42to0.43), while maintaining comparable median EF_10%_ across both targets (EGFR: ≈ 1.6to1.75; ECF-T: ≈ 2.4). In contrast, the ECR method achieved the lowest Spearman correlation for EGFR (*ρ* ≈ 0.21), but remained comparable to the other two scores for ECF-T (*ρ* ≈ 0.40), with a median EF_10%_ similar to Z-Score across both targets (EGFR: ≈ 1.5; ECF-T: ≈ 2.4), as shown in Fig. 2.

Computational cost, measured as runtime per compound (described in the Methods “Evaluation metrics”), was broadly comparable across Z-Score, RbR, and ECR for both targets, with median values ranging from ≈ 440 to 590 s/compound (Fig. 2, right panel). For EGFR, Z-Score and RbR exhibited nearly identical median costs (≈550 s/compound), while ECR yielded a notably lower median (≈440 s/compound). For ECF-T, this trend was reversed: ECR incurred the highest median cost (≈590 s/compound), whereas Z-Score was the most economical (≈485 s/compound). Overall, Z-Score and RbR consistently achieved the highest Spearman correlations within the top 1% of pipelines, while maintaining moderate computational costs, providing the most reliable balance between accuracy and efficiency. ECR consistently produced the weakest correlations, particularly for EGFR, despite offering no systematic cost advantage.

The spread of consensus methods across all evaluated pipeline results underscores the importance of systematic pipeline selection. For the EGFR target, Spearman correlations among the top 1% of pipelines ranged from *ρ* ≈ 0.21 (ECR) to *ρ* ≈ 0.37 (Z-Score and RbR), while for ECF-T all three methods converged more closely, spanning from *ρ* ≈ 0.40 to *ρ* ≈ 0.43. These differences illustrate that an uninformed choice of docking tool, SF, or consensus method can substantially degrade enrichment performance, reinforcing the need for a data-driven framework such as MolDockLab to identify the most effective workflow for a given target.

### Systematic pipeline analysis and selection

Having characterized individual components, we next evaluated the resulting complete docking pipelines to identify combinations that yield optimal ranking performance while maintaining computational efficiency. This analysis provides both mechanistic insight into high-performing configurations and the basis for automated pipeline selection.

**Fig. 3:**
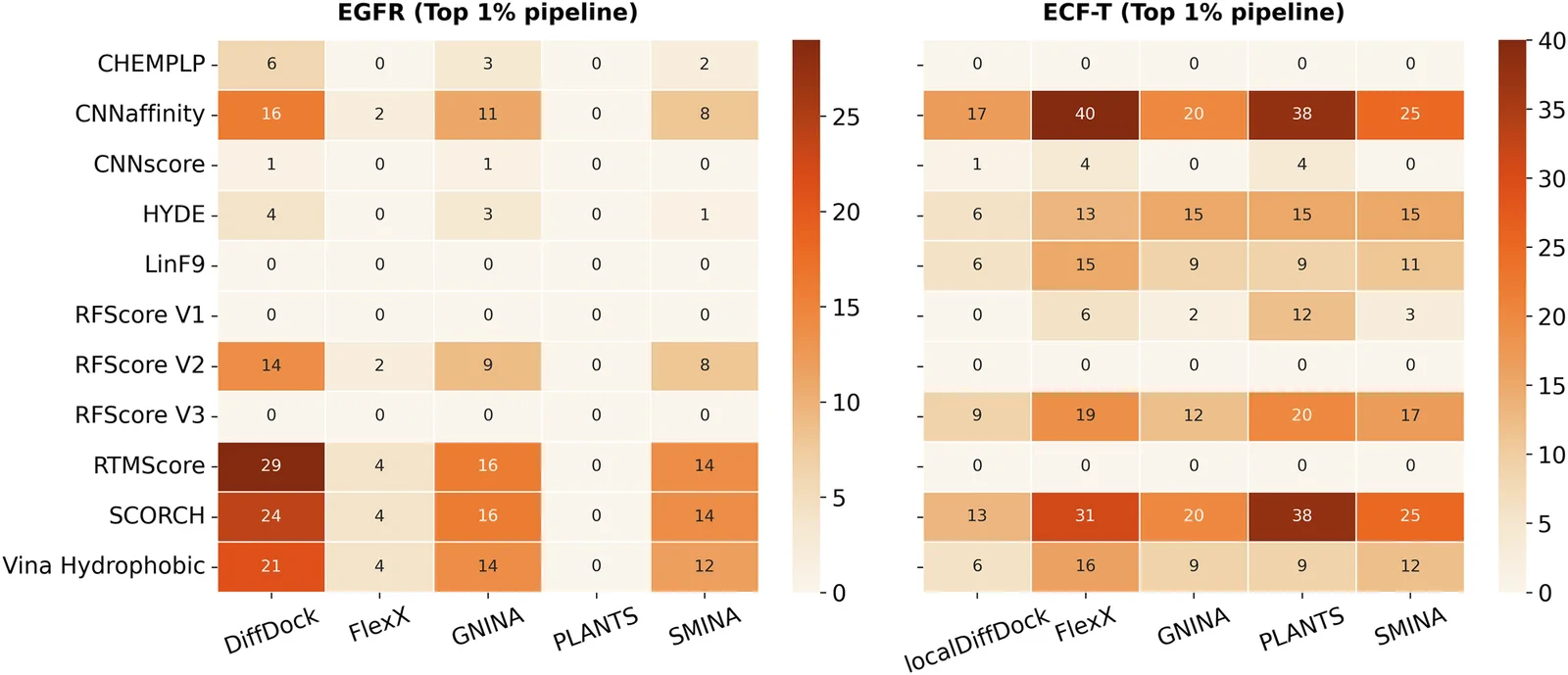
Frequency of docking tools and scoring functions (SFs) among the top 1% of pipelines for both targets. Distribution of docking-tool and scoring-function pairs within the top 1% of pipelines ranked by Spearman correlation, shown for the epidermal growth factor receptor (EGFR; left; *n* = 2853 pipelines) and the energy-coupling factor transporter (ECF-T; right; *n* = 5709 pipelines). Cell shading indicates how often each docking-tool and scoring-function combination occurs in the top-ranked subset, with darker shading denoting higher frequency.

To identify the most effective combinations of docking tools and SF, we analyzed the top 1% of pipelines ranked by Spearman correlation for each target. Figure 3 presents the frequency of each docking tool, SF pair within this top-performing subset for EGFR and ECF-T. For EGFR, DiffDock emerges as the dominant docking tool, consistently pairing with RTMScore (29), SCORCH (24), and Vina Hydrophobic (21) as the most frequently occurring SF. GNINA and SMINA also contribute meaningfully, particularly with RTMScore and SCORCH, while FlexX showed minimal representation and PLANTS none in the top-performing pipelines. In contrast, the ECF-T target is more evenly distributed across docking tools, with FlexX and PLANTS achieving substantial counts, notably PLANTS with SCORCH (38) and CNNaffinity (38), FlexX paired with CNNaffinity (40) and SCORCH (31). localDiffDock, while present, does not dominate as its counterpart does for EGFR, indicating that classical docking tools may be better suited for the ECF-T target.

These patterns reveal strong target dependence in optimal configurations, motivating an automated selection strategy tailored to each system. As described in “Pipeline selection”, MolDockLab provides an automatic selection procedure, i.e., it follows a user-defined tolerance-window for Spearman correlation and EF_10%_, with default tolerance values of 0.1 and 0.5, respectively. If there is more than one pipeline fulfilling the criterion, the one with the lower cost is chosen by default, offering the best trade-off between predictive performance and computational cost for each target. For the EGFR target, no pipeline fell within the default tolerance range, so the EF_10%_ tolerance was relaxed to 1. The selected pipeline pairs DiffDock for pose generation with RTMScore as the sole SF. This pipeline achieved *ρ* = 0.4602 and an EF_10%_ of 1.940, effectively on par with the highest-correlation pipeline (*ρ* = 0.4604, a difference of only 0.0002), while reducing computation time by roughly 1.2s per ligand. Because only each ligand’s top-scoring pose is retained, no consensus ranking step is required. For the ECF-T target, the default tolerance values (*ρ* = 0.1, EF_10%_ = 0.5) were used. The selected pipeline employs PLANTS for docking, with CNNaffinity, SCORCH, and RF-Score V3 as SF, and Z-Score for consensus ranking. Compared to the highest-correlation pipeline (*ρ* = 0.452, EF_10%_ of 2.58), this configuration achieved *ρ* = 0.447, while improving the EF_10%_ to 3.13 and saving on average 3 s per compound (runtime of 63.12s per compound). In both cases, the pipeline selection strategy successfully identified near-optimal configurations at reduced computational cost. These selected pipelines were subsequently used for the large-scale screening experiments. The full list of pipelines within the tolerance range for both targets are provided in Supplementary Tables 3 and 4.

The resulting target-specific pipelines were then compared against alternative modeling strategies to contextualize their performance. Having established the MolDockLab-selected pipeline for EGFR, we next asked how this combinatorial, docking-based strategy compares to a single co-folding model. To this end, we benchmarked the open-source co-folding model Boltz-2 on the same EGFR calibration set. Boltz-2 yielded a Spearman correlation of *ρ* = 0.134 between its confidence score and the experimental pIC_50_values, with an EF_10%_ = 1.14. As summarized in Table 3, these values are substantially below those obtained by the MolDockLab-selected pipeline on the same calibration set (*ρ* = 0.46, *EF*10% = 1.94), and approach random performance for the early-recognition metrics that are most relevant to virtual screening. Given this calibration-set performance, and given that scaling Boltz-2 to the full screening library would require GPU resources well beyond the modest CPU workstation sufficient for the MolDockLab-selected pipeline, we did not extend the Boltz-2 evaluation to the full screening library.

**Table 3:** Comparison of the MolDockLab-selected pipeline, and the Boltz-2 co-folding baseline on the epidermal growth factor receptor (EGFR) calibration set (*n* = 201)

	MolDockLab-selected	Boltz-2
Spearman *ρ*	0.46	0.13
EF_10%_	1.94	1.14
Runtime (s/compound)	411.6 (CPU)	203.4 (GPU A100)

The Boltz-2 runtime was measured on an NVIDIA A100 GPU and is not directly comparable to the CPU-only runtimes of the other pipelines (see the Methods, Hardware and runtime for the Boltz-2 baseline).

### Pipeline generalization and retrospective EGFR case study

To assess whether the selected pipelines generalize beyond the calibration data, we examined their performance on unseen chemical space using both retrospective screening and scaffold-based cross-validation. This analysis evaluates the robustness of pipeline ranking and its dependence on chemical diversity.

The EGFR screening set, comprising 6473 compounds, of which 5991 compounds were successfully docked (7.8% undocked). The resulting poses were rescored using RTMScore, followed by best-pose selection.

To assess whether optimizing for ranking quality or early enrichment yields better screening outcomes, we compared two selected pipelines. The correlation-selected pipeline (DiffDock + RTMScore) achieved *ρ* = 0.4602 and an EF_10%_ of 1.940 on the calibration set, while the EF-selected pipeline (DiffDock + CNNaffinity, HYDE, RFScore V2 with ECR ranking) achieved an EF_10%_ of 2.522 but a lower *ρ* = 0.304. When applied to the screening set, the correlation-selected pipeline outperformed the EF-selected one across all enrichment thresholds (1%, 3%, 5%, 10%), indicating that, for EGFR, calibration-set ranking quality translates more reliably to unseen data (see Table 4).

**Table 4:** Comparison of pipeline selection strategies on the epidermal growth factor receptor (EGFR) target

Metric	Correlation pipeline		EF pipeline		Naïve baseline	
	Screening set	Calibration set	Screening set	Calibration set	Screening set	Calibration set
Spearman correlation	0.36	**0.46**	0.19	0.30	0.01	0.03
EF_1%_	**2.22**	–	1.17	–	0.88	–
EF_3%_	**1.91**	–	0.98	–	0.89	–
EF_5%_	**1.720**	–	1.042	–	0.92	–
EF_10%_	1.57	1.94	1.06	**2.52**	0.94	1.03

The correlation-selected and enrichment factor (EF)-selected pipelines were identified via MolDockLab’s calibration set workflow. The naïve baseline reports the mean across all five docking engines (DiffDock, FlexX, GNINA, PLANTS, SMINA) using their native SFs without rescoring or consensus; per-tool values are provided in Supplementary Tables 1 and 2.

Bold values indicate the best result in each metric.

To further quantify the advantage of a data-driven pipeline selection, we evaluated a naïve single-tool baseline averaged across all five docking engines (DiffDock, FlexX, GNINA, PLANTS, SMINA), each using its own native SF without informed rescoring or consensus. On the calibration set, this baseline yielded a mean Spearman correlation of *ρ* = 0.03 ± 0.15 and a mean EF_10%_=1.03 ± 0.32. On the screening set, the same baseline yielded a mean Spearman correlation of *ρ* = 0.01 ± 0.17 and mean enrichments of EF_1%_=0.88 ± 0.27, EF_3%_=0.89 ± 0.24, EF_5%_=0.92 ± 0.17, and EF_10%_=0.94 ± 0.14. Performance varied substantially across tools, with screening-set Spearman correlations ranging from *ρ* = − 0.135 (DiffDock) to *ρ* = 0.303 (GNINA), showing that the performance of any single tool is highly variable and, on average, substantially worse than the correlation- or EF-selected pipelines from MolDockLab. The full per-tool breakdown is provided in Supplementary Tables 1 and 2. All three configurations are compared in Table 4. Together, these results demonstrate that optimization based on ranking quality translates effectively to prospective enrichment and outperforms naïve or heuristic approaches.

**Fig. 4:**
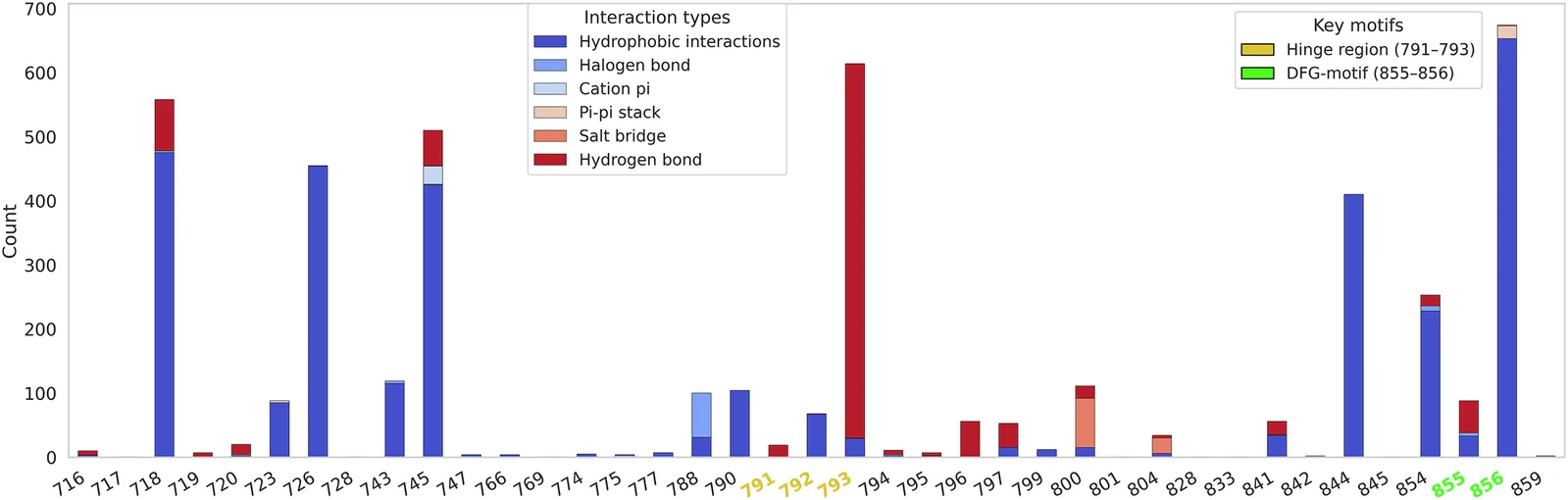
Interaction hot-spot analysis of active-compound poses in the epidermal growth factor receptor (EGFR) binding site. Frequency of each interaction type per residue across 510 DiffDock-generated poses of active compounds in the EGFR binding site. Each stacked bar represents one residue, and the coloured segments give the count of each interaction type at that residue. Hinge-region residues 791 to 793 are shaded in yellow and aspartate--phenylalanine--glycine (DFG) motif residues 855 to 856 are shaded in green.

To further examine whether structure-based criteria, i.e., interaction hotspots, can refine ranking-based selection, we next incorporated an interaction-based filtering step using PLIP, focusing on key binding-site contacts in the EGFR kinase domain. Key residues were identified by generating ligand poses with DiffDock for a set of 51 known active ligands retained from the calibration set with pIC_50_≥ 8. The two most frequently observed interactions involved the MET793 residue, located in the hinge region and typically functioning as a hydrogen bond, and the PHE856 residue, part of the DFG-motif, which is predominantly engaged in hydrophobic contacts. Notably, all active poses formed interactions with MET793 in the hinge region and with PHE856 in the DFG-motif, as depicted in Fig. 4, underscoring their critical role in ligand binding.

To pass the filtration criteria, each compound was required to have at least one pose, among its ten generated poses per docking tool, that formed an interaction with either MET793 or PHE856. Conversely, compounds for which none of the generated poses exhibited an interaction with either of these two key residues were discarded from further consideration. These two residues were selected because they anchor the two pharmacologically most relevant regions of the EGFR kinase binding site: the hinge region, which mediates the critical hydrogen bond to the adenine moiety of ATP and most known inhibitors, and the DFG-motif, whose conformation discriminates active from inactive kinase states.

This filtering process resulted in 5823 compounds out of 5991 docked compounds through DiffDock. Among the filtered compounds, only 12 were experimentally active (pIC_50_≥8); the remaining 156 were classified as inactive (Table 5). This filtration marginally increased the proportion of active compounds from 25.5% to 26.1%. The corresponding pIC_50_distributions before and after filtering are provided in Supplementary Fig. 7 in the Supplementary Information.

**Table 5:** Confusion matrix for the PLIP-based interaction filter applied to 5991 docked epidermal growth factor receptor (EGFR) compounds

	Active	Inactive
Retained	1520	4303
Removed	12	156

Rows indicate filter outcome (retained or removed); columns indicate experimental activity status (pIC_50_≥ 8).

The PLIP-based filter achieved a sensitivity of 99.2%, retaining nearly all active compounds, with only 12 actives lost out of 1532. However, specificity remained low (3.5%), and the F1-score of 41.3% reflects the limited ability of a two-residue criterion to deplete inactive compounds. This is expected, as MET793 and PHE856 are high-contact residues that most docked poses naturally engage, rendering the filter too permissive to act as an effective classifier. Nevertheless, the value of this step lies not in its discriminative power but in its role as a physically grounded sanity check: by requiring at least one interaction with a pharmacologically essential anchor residue, the hinge-region (MET793) or the DFG-motif (PHE856), the filter ensures that only compounds adopting a plausible binding mode are carried forward. We expect that the effectiveness of this step will scale with the quality of the selected anchor residues; for targets where well-defined, selective pharmacophoric anchors can be identified, this criterion should provide a substantially more stringent filter.

**Fig. 5:**
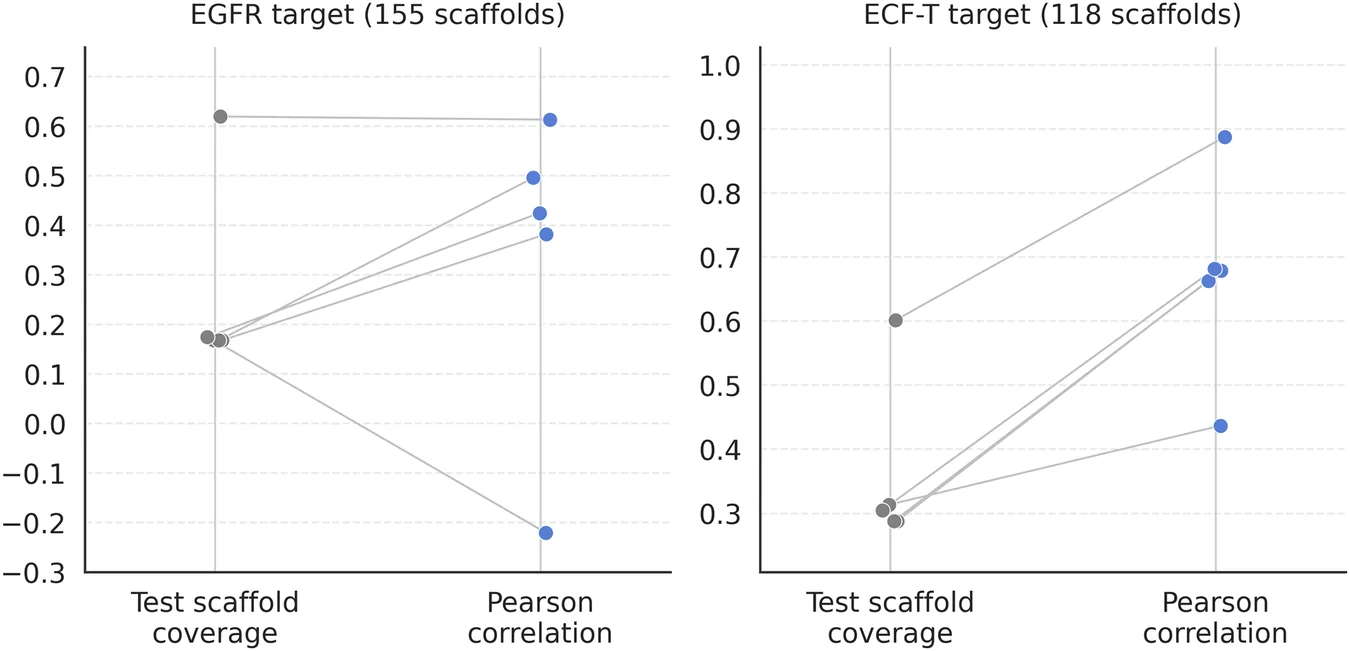
Relationship between calibration-set scaffold coverage and the transfer of pipeline rankings from training to test splits. Five-fold scaffold-based cross-validation for the epidermal growth factor receptor (EGFR; left) and the energy-coupling factor transporter (ECF-T; right). For each fold, the gray marker gives the test scaffold coverage (the fraction of unique scaffolds in the test split relative to all unique scaffolds in the calibration set) and the blue marker gives the Pearson correlation between the pipeline rankings obtained on the training and test splits; a connecting line links the two values for the same fold. Higher scaffold coverage is associated with stronger train-to-test ranking transfer.

Beyond these target-specific analyses, we next assessed whether the observed pipeline rankings generalize to unseen chemical space. To evaluate whether pipeline rankings were trained on the calibration set transfer to unseen compounds, we performed 5-fold scaffold-based cross-validation described in section “Generalizability assessment”. For each fold, we correlate the ranking of pipelines across both training and test set. This way, we assess how much the pipeline ranking is influenced by the calibration set. Additionally, we report the test scaffold coverage, as the fraction of unique scaffolds in the test set to the total number of unique scaffolds in the full calibration set. In Fig. 5, we report the pipeline performance Pearson correlation between training and test pipeline rankings alongside scaffold coverage.

As shown in Fig. 5, the degree to which pipeline rankings transfer from training to test splits is closely linked to scaffold coverage. For the EGFR target (total of 155 unique scaffolds), fold 1, which captures the largest share of the calibration set’s scaffolds (≈0.62), achieves the highest correlation (*r* ≈ 0.61). As scaffold coverage decreases across the remaining folds, correlation declines accordingly, with fold 2 a notable outlier (*r* ≈ − 0.22), likely due to a highly active scaffold absent from its training set. A similar trend is observed for the ECF-T target (total of 118 unique scaffolds). Fold 1, with the highest coverage (≈0.60), yields the strongest correlation (*r* ≈ 0.89), while folds 3,4 and 5, at lower coverage (0.29 to 0.31), show moderate correlations (0.66 to 0.68). Again, Fold 2 is an outlier (*r* ≈ 0.44) despite comparable coverage, pointing to a similar scaffold-driven effect.

These results highlight that pipeline selection is most reliable when the calibration set adequately represents the scaffold diversity of the intended screening library. When the calibration compounds span the relevant chemical space well, the selected pipeline transfers effectively to unseen compounds. In practice, this underscores the importance of assembling a calibration set that is chemically representative of the screening collection, a criterion that MolDockLab users can address by ensuring broad scaffold coverage in their initial bioactivity dataset.

### Prospective screening campaign: ECF-T case study

**Fig. 6:**
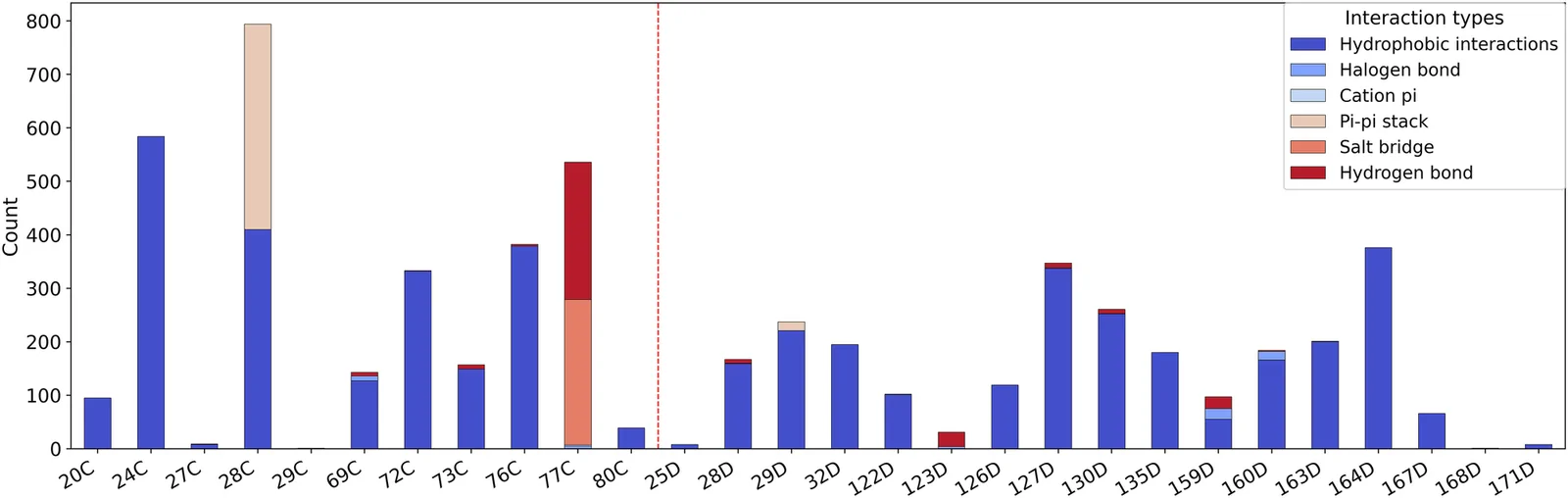
Interaction fingerprint of active compounds in the energy-coupling factor transporter (ECF-T) binding site. Frequency of each interaction type per residue, summarizing all contacts from 54 active ligands (ten poses each) in the ECF-T binding site. Each stacked bar represents one residue and the coloured segments give the count of each interaction type. Residues from chain C are plotted to the left of the central dashed line and residues from chain D to the right, each ordered by residue number.

Finally, we applied the MolDockLab in a prospective screening setting to demonstrate its practical utility for hit identification, refinement, and experimental validation. The selected optimal pipeline for ECF-T utilizes PLANTS for docking, CNNaffinity, SCORCH, and RF-Score V3 for rescoring, along with Z-Score for consensus ranking. It achieved a Spearman correlation of 0.447, just 0.005 less than the leading workflow. It enhanced EF_10%_ to 3.13 compared to the top-ranked pipeline 2.58, with a runtime of 63.12s per compound, saving 3s per compound. This pipeline was subsequently applied to the in-house HIPS collection of 6505 molecules. PLANTS produced valid docking poses for 6235 compounds, while no good pose could be calculated for the remaining molecules. All valid poses were rescored and ranked, and the highest-ranked top 1% compounds were carried forward to post-processing for hit selection. To further refine the hit selection, protein–ligand interactions were again analyzed using PLIP. We started with 54 active compounds from the calibration set, whose poses were generated using PLANTS. PLIP analysis highlighted seven recurrent hot-spot residues within the binding site, LEU24, PHE28, LEU76 and ARG77 from the S-component and THR127, THR130 and LEU164 from the ECF-T interface, as shown in Fig. 6. Compounds interacting with at least six of these residues were retained, resulting in a refined subset representing the most favorable interaction motifs.

To identify a subset of potential hit candidates for experimental testing, we clustered the top hits with respect to structural similarity. Thus, the retained poses were clustered with K-medoids to maximize chemical diversity, and picking the cluster centers yielded 11 representative compounds. Compounds **11** and **16** were additionally included due to their high scores relative to those of the selected cluster centers. Expert visual inspection identified four additional scaffolds (compounds **3,** **4,** **5**, and **6**), that were considered pharmacologically interesting but under-represented in the clustering outcome. The combined selection of 17 candidates, depicted in Supplementary Fig. 8, was advanced to in vitro validation.

**Fig. 7:**
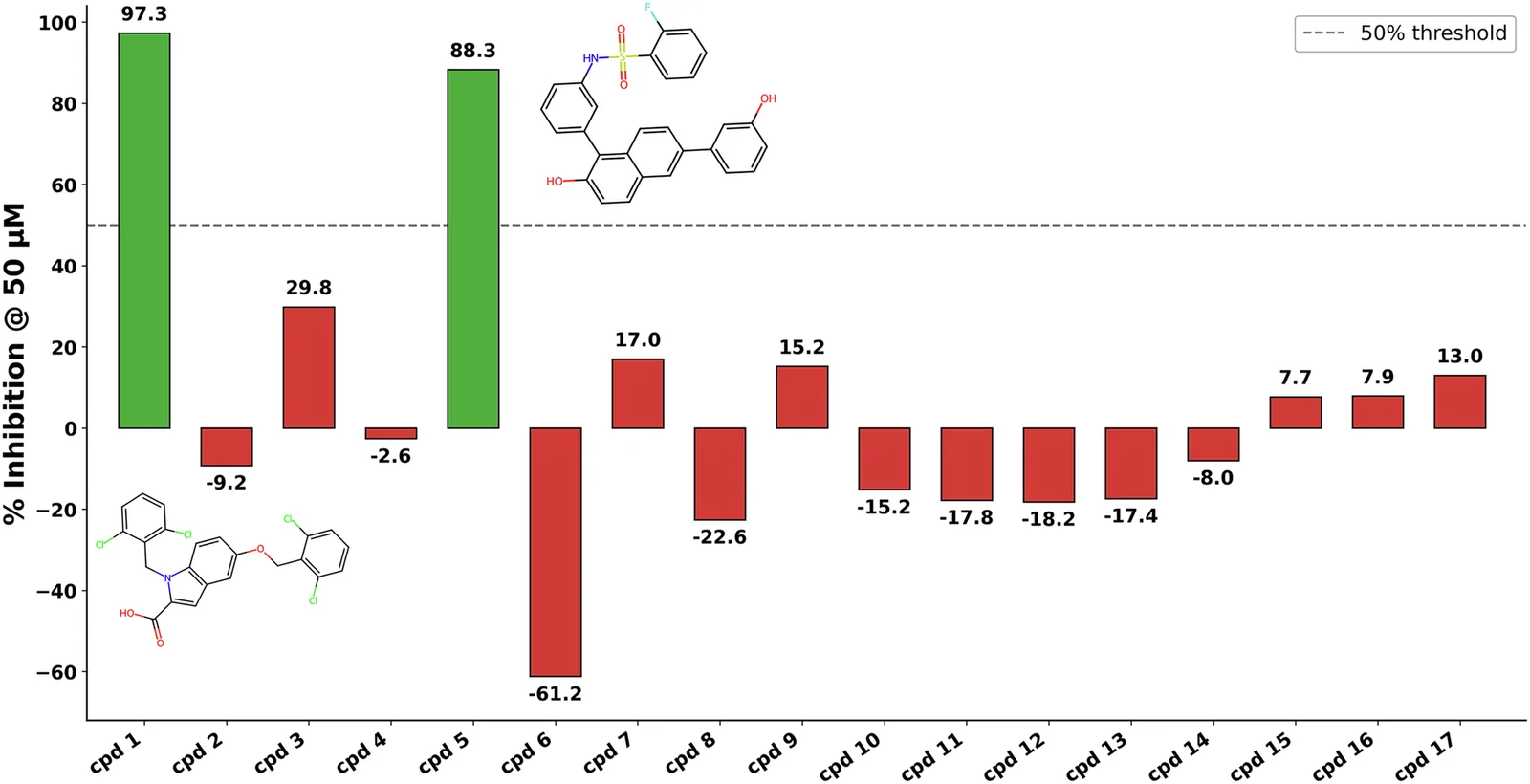
Whole-cell inhibitory activity of the seventeen MolDockLab-selected compounds at a single concentration. Percentage inhibition of compounds **1** to **17** measured at 50 micromolar in the whole-cell uptake assay. Green bars indicate compounds exceeding 50% inhibition (the dashed horizontal line marks the 50% threshold); red bars indicate inactive or negatively active compounds. The chemical structures of the two active compounds are shown below the plot (compound **1**) and to the right of the plot (compound **5**).

Following filtering, diversity clustering and manual inspection, the final compound set was advanced to experimental validation. All 17 candidates were evaluated for their inhibitory activity in a whole-cell uptake assay using *Lactobacillus casei*, as a Gram-positive non-pathogenic model organism^68^, more details in the Methods (Whole-cell bacterial uptake assay). All compounds were initially evaluated at a single concentration of 50 *μ*M. Two compounds from the MolDockLab-selected set exhibited high inhibitory activity at this concentration: compound **1** (97.3%) and compound **5** (88.3%), as shown in Fig. 7. These two hits were subsequently subjected to dose-response assays, yielding IC_50_values of 11.8 *μ*M and 18.1 *μ*M, respectively. Notably, these values are within the same order of magnitude as the most potent ECF-T inhibitor reported to date, which exhibits single-digit micromolar activity, highlighting the effectiveness of the MolDockLab-driven selection for this challenging target.

**Fig. 8:**
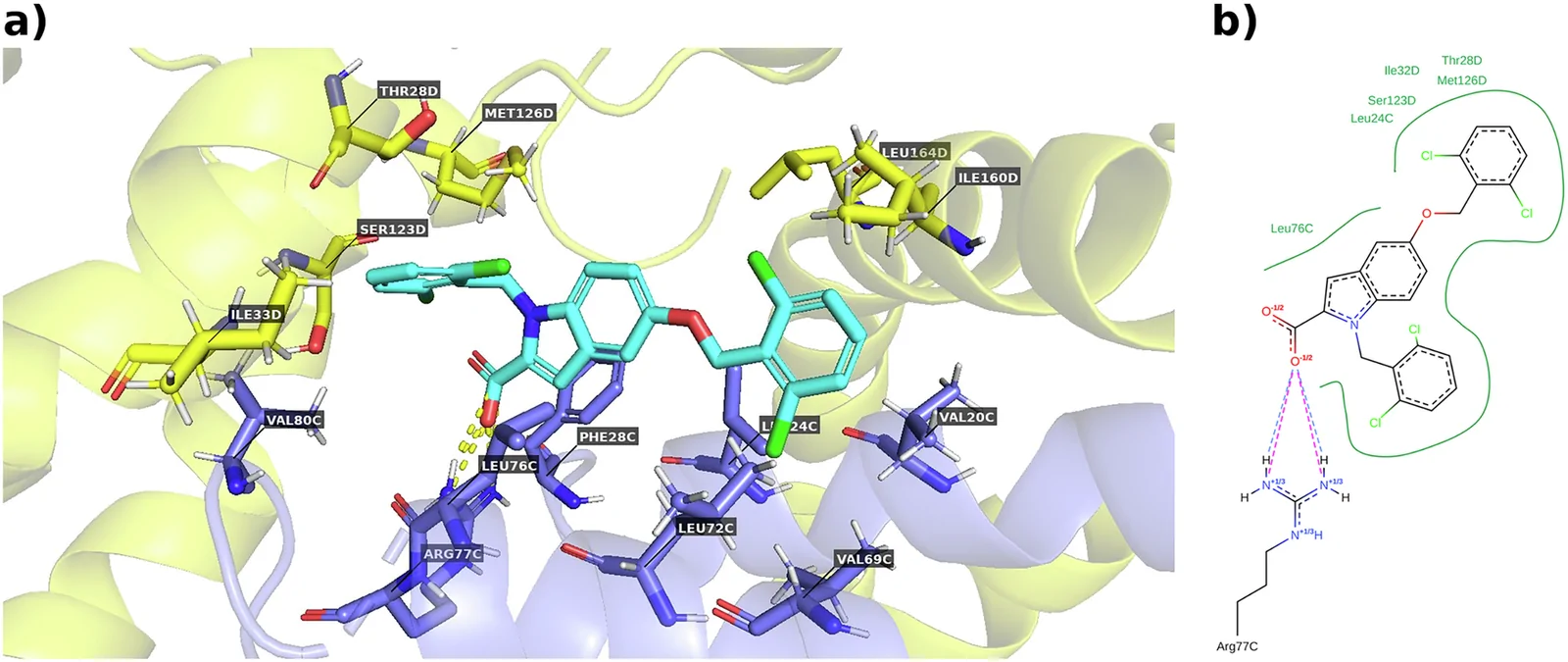
Predicted binding mode of compound **1** at the interface of the energy-coupling factor transporter. Binding analysis of the compound **1** pose generated with PLANTS, located at the interface between the S-component (chain C, blue) and the EcfT component (chain D, yellow). **a** Three-dimensional structural view rendered in PyMOL^84^, showing compound **1** (cyan) within a predominantly hydrophobic pocket formed by residues from both chains and forming a hydrogen bond with arginine 77 of chain C. **b** Two-dimensional interaction diagram generated with PoseEdit^85^: hydrophobic contacts are shown as green contours, hydrogen bonds as blue, dashed lines, and the salt-bridge interaction with arginine 77 of chain C as magenta, dashed lines.

The predicted binding mode of compound **1**, corresponding to the top-ranked pose generated by PLANTS based on the best Z-Score, is depicted in Fig. 8. The compound binds at the interface between the S-component (chain C) and EcfT interface (chain D), occupying a predominantly hydrophobic cavity. The carboxylate moiety of compound **1** forms a salt bridge with the guanidinium group of Arg77C, with a distance of approximately 2.5 Å, representing the key polar interaction anchoring the ligand within the binding site. Additional contacts with Ser123D and Thr28D further stabilize the polar region of the ligand. The dichlorophenyl moiety is oriented toward a hydrophobic subpocket lined by Leu24C, Leu76C, and Ile32D, while the central aromatic core engages in van der Waals interactions with Val80C, Met126D, and Leu164D. The overall binding pose positions the ligand across the subunit interface, consistent with a mechanism of action that disrupts the conformational dynamics required for substrate translocation.

**Fig. 9:**
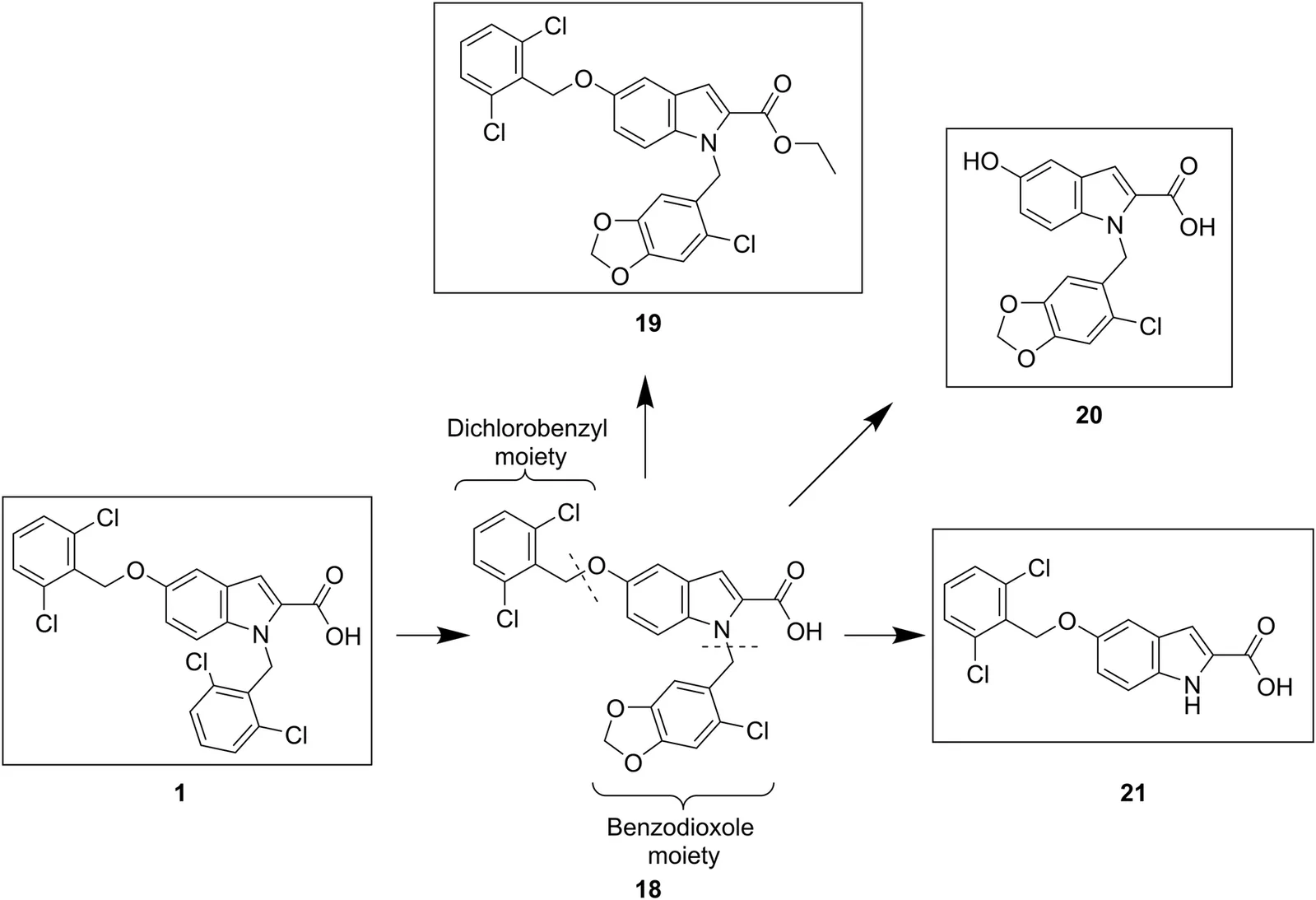
Structure–activity relationship series derived by truncating the optimized compound 18. Compound **1** (bottom left) was optimized to compound **18** (center), which was then truncated to assess the contribution of each structural moiety. Compound **20** lacks the dichlorobenzyl moiety, compound **21** lacks the 6-chloro-1,3-benzodioxole moiety, and compound **19** is the corresponding ethyl ester of compound **18**. Bold numbers denote the compounds tested in the whole-cell uptake assay.

To further explore the chemical determinants of activity, we performed a focused structure–activity relationship (SAR) analysis on the most promising hit. Thus, a similarity search for the close analogues of compound **1** in our in-house library led to the identification of compound **18**, shown in Fig. 9. Compound **18** showed potent inhibition of folic acid uptake in the whole-cell uptake assay, with a percentage inhibition of 90.4% at 50 *μ*M and an IC_50_value of 6.9 *μ*M.

Based on this promising activity, a concise SAR study was performed to explore the structural features essential for activity. Essentiality of the carboxylic acid group was evaluated by testing the equivalent ester (compound **19**), and a truncation strategy was employed by systematically modifying dichlorobenzyl and 6-chloro-1,3-benzodioxole moieties of compound **18** to evaluate their effect on inhibitory activity, as illustrated in Fig. 9. Selective removal of the dichlorobenzyl moiety (compound **20**) completely abolished the activity, indicating its critical role in target engagement. On the other hand, removal of the 6-chloro-1,3-benzodioxole moiety (compound **21**) while retaining the dichlorobenzyl moiety significantly reduced activity, with only 17.8% inhibition compared to the 90.4% observed for the parent compound (compound **18**). These findings highlight the importance of both regions in achieving potent inhibitory activity. Furthermore, the presence of the ester group (compound **19**) completely abolished the activity, indicating the free acid’s critical role in inhibitory activity. The experimentally confirmed hits validate the end-to-end workflow, demonstrating that the proposed strategy can be reliably deployed in virtual screening campaigns targeting other biological systems. This is particularly compelling given the inherent difficulty of the ECF-T target, a transmembrane protein with a cryptic, membrane-embedded binding site, no co-crystallized ligand, and no previously known inhibitors, yet the workflow delivered hits with IC_50_values in the low micromolar range, comparable to the most potent ECF-T inhibitors reported to date.

## Discussion

SBVS remains pivotal for hit discovery, yet pose accuracy and binding-affinity ranking still limit its reliability. We present MolDockLab, a data-driven framework that systematically evaluates all combinations of five docking tools, 15 SF, and three consensus-ranking strategies on a small calibration set of compounds with known bioactivity, then selects the optimal pipeline for a given protein target rather than enforcing a generic workflow.

Retrospective validation on EGFR demonstrated that the correlation-selected pipeline (DiffDock + RTMScore) achieved a Spearman correlation of 0.36 on the screening set and EFs exceeding those of both the EF-optimized and naïve baseline pipelines across all thresholds. Notably, the naïve single-tool baseline averaged across all five docking engines yielded near-zero Spearman correlations on the screening set (*ρ* = 0.01 ± 0.17) and mean EFs at or below 1.0 across all thresholds, underscoring the substantial benefit of informed, data-driven pipeline selection.

In the prospective ECF-T case study, the selected pipeline (PLANTS + CNNaffinity, SCORCH, and RF-Score V3 with Z-Score ranking) reached a Spearman correlation of 0.45 and an EF_10%_ of 3.13 on the calibration set. Subsequent post-processing through PLIP-based interaction filtering, K-medoids diversity clustering, and expert visual inspection identified 17 candidates for in vitro testing. Of these, two compounds exhibited strong inhibitory activity (97.3% and 88.3% inhibition at 50 *μ* M), with IC_50_values of 11.8 *μ* M and 18.1 *μ* M, respectively. A follow-up similarity search further uncovered compound **18** (IC_50_= 6.9 *μ* M), and an initial SAR study delineated essential pharmacophoric features, confirming that MolDockLab can deliver chemically novel, experimentally validated hits. The prospective discovery of low-micromolar ECF-T inhibitors, achieved without a co-crystallized ligand against a transmembrane target with a dynamic, membrane-buried binding pocket, illustrates that MolDockLab can deliver experimentally validated hits even for target classes traditionally considered out of reach for structure-based methods.

The cross-validation analysis revealed that pipeline generalizability is closely tied to scaffold coverage in the calibration set, highlighting the importance of assembling a chemically representative calibration library. This observation points toward several promising directions for future development. Expanding the panel of SFs to include physics-based and knowledge-based approaches could further improve ranking fidelity. As the number of tools grow, an exhaustive evaluation of all combinations will become infeasible. The current work can serve as a basis for exploring trade-offs of combinatorial search strategies. Evaluating the workflow on larger and more diverse benchmark datasets, such as LIT-PCBA, will help establish its scalability across chemical libraries of varying size and composition. Additionally, investigating how structural and functional similarity between protein targets influences optimal pipeline selection could enable transfer-learning strategies that reduce the dependence on target-specific calibration data, extending MolDockLab’s applicability to novel targets with limited experimental information.

Taken together, MolDockLab advances SBVS by formalizing target-specific optimization in a reproducible, largely automated framework, with expert visual inspection retained as a final step. By bridging the gap between exhaustive combinatorial benchmarking and practical hit identification, MolDockLab provides the computational chemistry community with a versatile tool that enhances both the efficiency and the success rate of early-stage drug discovery campaigns.

## Methods

We propose MolDockLab as a SBVS workflow tailored for targets where some inhibition data is available. This information is systematically leveraged to identify the most predictive virtual screening pipeline prior to large-scale screening. An overview of the complete workflow is provided in Fig. 1.

At its core, the workflow introduces a strategy for selecting an optimal combination of methods for future SBVS campaigns. The choice of such a combination is a critical aspect of any virtual screening effort, and MolDockLab aims to provide a comprehensive and systematic solution for its identification. To this end, the workflow performs exhaustive evaluations across five docking algorithms, 15 SF, and three consensus ranking methodologies, yielding a total of 3,047,361 possible pipelines. All combinations are assessed on the initial input data to determine the pipeline that performs best with respect to the available experimental measurements. In the following sections, we describe the individual steps such as ligand-library preparation, docking, rescoring, consensus ranking, pipeline selection, data post-processing, and hit selection.

### Datasets and target preparation

For evaluating MolDockLab, we selected two biological systems that cover complementary scenarios. The first target, EGFR, is a well-characterized kinase with abundant experimental data, providing a benchmark for retrospective validation. The second target, ECF-T, is a transmembrane transporter with a cryptic binding site, limited experimental data, and no known inhibitors, representing a challenging prospective application that tests the workflow under realistic drug discovery conditions.

The epidermal growth factor receptor (EGFR) (UniProt ID P00533), is a thoroughly researched human protein kinase, with 383 structures recorded in the protein data bank (PDB) as of 12th March 2026^69^. We employed the following inclusion criteria to choose a suitable protein structure: a structural resolution better than 3 Å, an entry consisting of a single chain, and the presence of a co-crystallized ligand with a molecular mass exceeding 100 Da. From the 225 crystal structures retrieved, we chose the protein structure with PDB ID 5UG9, resolved to 1.33 Å, whose co-crystallized ligand interacts with the kinase hinge region and the DFG-in motif. The DFG-in conformation was selected because Type I (ATP-competitive, DFG-in) binders dominate the reported EGFR inhibitor space, including all first- to third-generation clinical agents, whereas structurally validated Type II (DFG-out) EGFR ligands remain comparatively rare^70,71^. The present study is therefore scoped to Type I EGFR binders. The selected structure was refined to correct missing structural information such as tautomeric forms, torsional angles, and amino acid protonation states using Protoss^72^. All water and non-essential molecules were removed. The binding site was identified by residues located within a radius of 5 Å distance from any ligand atom.

As screening compound library, we retrieved 7287 EGFR ligands with reported bioactivity from ChEMBL33^73^. We discarded entries with missing values, duplicate compound ChEMBL identifiers (ChEMBL IDs), and activity measurements reported as endpoints other than IC_50_, resulting in 6687 compounds. To avoid data leakage, we excluded further 13 molecules whose ligand-protein complexes appear in DiffDock’s training dataset. Activity labels were assigned using pIC_50_values, defined as the negative logarithm (base 10) of the IC_50_values. Compounds with pIC_50_≥ 8.0 were labeled active (*n* = 1698), while the remainder, although still exhibiting measurable activity at lower potencies, were labeled inactive, or rather less active, for the purpose of this binary classification. The choice of pIC_50_≥ 8.0 reflects the well-explored nature of EGFR, for which thousands of sub-micromolar inhibitors are already annotated and only high-affinity binders are practically interesting as starting points for a novel kinase program; lowering the threshold to pIC_50_≥7.0 would result in a dataset containing ~ 49.4% actives, which is itself unrealistic for a virtual screening scenario. The active set retains substantial chemical diversity at this threshold, with a mean pairwise Tanimoto similarity of 0.21 (ECFP4, radius 2, 2048 bits). A full sensitivity analysis across activity thresholds is provided in Supplementary Fig. 1. A stratified random split by activity class then yielded a calibration set of 201 molecules and a screening set of 6473 molecules. The calibration subset served to select the SBVS workflow for the EGFR target, whereas the screening set was used to evaluate MolDockLab’s performance (Table 6).

**Table 6:** Summary of compound-selection and -classification for the EGFR and ECF-T targets

Description	EGFR	ECF-T
Initial compound pool	7287	6717
Compounds with experimental activity data	7287	212
Removing missing values and duplicates	7039	—
Excluding non-IC_50_activities	6687	—
Excluding ligands in DiffDock training set	6674	—
Calibration subset / Screening set	201 / 6473	212 / 6505

For the prospective study, an ECF-T structure for the pantothenate (ECFPanT) is published in its crystallized apo-form, with PDB ID 6ZG3, resolved at a resolution of 2.80 Å^74^. In prior work, an in-house inhibitor was identified as an active compound against ECF-Ts, with an IC_50_= (282 ± 108)*μ*M determined by an ECFPanT uptake assay in *Lactobacillus delbrueckii*^66^. As no experimental binding site was available, coarse-grained molecular dynamics (MD) simulations were performed using this structure as the starting point, revealing two potential pockets, P9 and P11^66^. Follow-up 60 nanosecond MD simulations, guided by three additional validated HIPS inhibitors, showed that pocket P9 remained markedly more stable than P11. Consequently, it was selected as the operative binding site for subsequent studies^67^. For the present study, a representative snapshot from these MD simulations was extracted and prepared using Protoss^72^, following the same protocol as described for EGFR, to serve as the protein structure for docking.

As screening library, potential ECF-T ligands were taken from the in-house HIPS compound library. The library contains 6717 compounds, with 212 possessing experimental values for the ECF-T target, making them suitable for use as MolDockLab’s calibration set, while the remaining 6505 compounds were used as the screening set (Table 6). Only 38 out of the 212 compounds had IC_50_values, whereas the remaining 174 compounds only had inhibition data at a single concentration or a reduced set of concentrations, complicating comparisons within the calibration set. To harmonize these heterogeneous endpoints, we applied Hodge ranking^75^ to produce a single consistent ranking. This ranking is synthesized from pairwise comparisons between measurements, where comparable measurement types are given. Concretely, pairwise differences between compounds are computed: inhibition comparisons are weighted by measurement confidence and concentration, while IC_50_comparisons contribute an additional global signal scaled by a bonus factor. These comparisons form a weighted, directed graph where edges encode relative preference strengths. Using the method of statistical ranking based on combinatorial Hodge theory^75^, the graph is decomposed via a graph Laplacian, and a global score for each compound is obtained by solving a least-squares problem that best fits all pairwise differences while removing cyclic inconsistencies. The resulting scores provide a consistent ranking that integrates both inhibition and IC_50_evidence on a unified scale.

For the 38 compounds with experimentally measured pIC_50_values, we compared their calculated scores to these potencies. Supplementary Fig. 2 shows a strong negative correlation between the two, indicating that the scoring scheme is predictive.

For both libraries, we utilized Gypsum-DL to predict a single 3D starting conformation for each compound, considering variations in ionization states within a pH range of 6.5 to 7.5, as well as tautomeric, chiral, cis-trans isomeric, and ring-conformation changes^76^. When Gypsum-DL failed to produce a pose, we turned to RDKit^77^ to generate ten random conformers following the addition of hydrogen atoms. The energy for each conformer was evaluated using the Merck Molecular Force Field (MMFF94)^78^, and the conformer with the lowest energy was selected as the recommended initial pose for the compound.

For 3D ligand generation, the EGFR target calibration set comprised 201 compounds, with 200 initial 3D structures generated using Gypsum-DL and one compound using RDKit. While, the initial 212 3D-compounds for the ECF-T target were prepared entirely using Gypsum-DL.

### Docking pose generation, scoring and pipeline selection

MolDockLab employs a consensus docking strategy that combines five docking tools to broadly sample the ligand pose space. Each engine generates ten poses per ligand, yielding up to 50 candidate poses in total. The consensus is formed by rescoring all 50 poses with a panel of SFs and aggregating their rankings through multiple consensus-based approaches, thereby reducing the bias inherent to any single docking engine or SF.

The pose generation stage draws on five docking engines, each run within MolDockLab as described below. The five docking engines deployed, DiffDock v1.0^24^, PLANTS v1.0^9^, FlexX v6.0.0^20^, SMINA^40^, and GNINA v1.0^48^, span distinct search strategies. Of these, FlexX is a commercial product distributed by BioSolveIT GmbH, while DiffDock, PLANTS, SMINA, and GNINA are open-source. Note that MolDockLab remains fully operational without integration of commercial software, and users without a FlexX license can run the workflow with the four open-source docking engines. All programs were run with their default settings. The individual output files, containing the coordinates of the docked poses, were concatenated into a single file for downstream rescoring and analysis.

DiffDock v1.0 was parameterized to stay close to a user-defined pocket. The parameterized algorithm is available at https://github.com/mbackenkoehler/DiffDock, referred to as localDiffDock. In standard DiffDock, the ligand is placed randomly relative to the entire protein surface before the reverse diffusion process begins. In localDiffDock, user-supplied binding pocket residues are used to compute a pocket centroid from the corresponding C_*α*_ coordinates, and the ligand’s center of mass is translated to this centroid prior to the application of translational, rotational, and torsional noise. This biases the starting configuration towards the known binding site, effectively converting DiffDock’s blind docking behavior into a pocket-conditioned approach.

For the EGFR target, standard DiffDock was used, as it correctly identified the intended binding site. 13 ligands associated with co-crystallized structures present in the training dataset of the DiffDock model were excluded. For targets with cryptic cavities, such as the P9 of the ECF-T target, DiffDock exhibited limitations in accurately predicting the binding site. Therefore, we applied localDiffDock with the binding pocket defined as all residues within a 5 Å radius of the co-crystallized ligand. All parameters were kept at their default values, except for the maximum translational degree of freedom for the ligand, which was set to 0.5 in the model configuration to restrict the ligand to the binding pocket.

PLANTS v1.0 was run after converting both the protein structure and ligand libraries into MOL2 format using OpenBabel v3.1. 0 ^79^. The binding site center was determined by calculating the centroid of the reference ligand’s coordinates using PLANTS, with a binding site radius of 5.0 Å.

FlexX v6.0.0^20^ was deployed using default settings. The binding site was defined by the co-crystallized reference ligand in SDF format. All other parameters were kept at their default settings, which restrict six-membered saturated rings to low-energy chair conformations and preserves all stereo centers.

Both SMINA and GNINA were executed through gnina v1.0, which provides access to both docking modes. To perform SMINA docking, the cnn_scoring option was set to none, causing the program to rely solely on its empirical SF. For GNINA docking, cnn_scoring was set to general_default2018, enabling the CNNs to rescore and re-rank poses. In both cases, a fixed random seed was used to ensure reproducibility, the exhaustiveness was set to 32, and the binding pocket was defined by the co-crystallized reference ligand in PDB format.

MolDockLab rescores the pooled docking poses with a diverse set of SFs spanning four machine-learning-based approaches (GNINA’s CNN-based scoring^48^, RTMScore^50^, SCORCH^49^, and RFScore^47^) and six empirical SFs (SMINA’s empirical^40^, Vinardo^41^, PLANTS’s CHEMPLP^9^, HYDE^42^, vina (intra)-hydrophobic^15^, and AD4^10^). Among these, HYDE is a commercial product distributed by BioSolveIT GmbH and requires a valid license, whereas all other SFs are openly available; as with FlexX in the docking stage, MolDockLab remains fully functional without it. Each SF was executed with its default settings using the prepared protein structure, the defined binding pocket, and the reference ligand as input. Full technical details and parameter listings are provided in the Supplementary Information (Technical details of scoring functions).

To reduce the combinatorial search space of downstream consensus pipelines, a SF selection step is applied. Pairwise Spearman correlations (*ρ*) are computed across all SFs using the pooled poses, and whenever two SFs correlate above a threshold of 0.9, the one with the weaker correlation between its predicted scores and experimental activity values is discarded. This can substantially reduce the number of retained SFs to keep only non-redundant scoring signals without losing complementary ranking information.

Before the retained SFs could be combined, their raw outputs had to be placed on a comparable scale. The HYDE SF presented a notably broad value range. A log-transformation followed by RobustScaler-scaling from ScikitLearn was initially evaluated but yielded a HYDE distribution centered around five, while all other SFs are centered around zero, and an order of magnitude wider than the other SF, which would cause HYDE to dominate any score-based consensus ranking. To address this, we applied a *rank-plus-cap* scheme: an upper threshold of 100,000 was applied to cap extreme values, and the rank of each compound among the HYDE scores was added to the threshold. For example, if a compound ranked 5th among all HYDE scores and its raw value exceeded 100,000, it was assigned a value of 100,005. This ensured that ranking information was retained while controlling the scale of the values, preventing HYDE from dominating other SFs in downstream consensus calculations.

To place all SFs on a common “higher-is-better” scale, we inverted those functions which report the estimated binding affinity as the Gibbs free energy Δ*G*, namely SMINA affinity, AD4, LinF9, Vinardo, CHEMPLP, HYDE, Vina-hydrophobic, and intra-hydrophobic. The resulting values were then robustly standardized with RobustScaler from scikit-learn, which rescales data according to the median and the 5th/95th percentiles and, thus, dampens the impact of individual SF and extreme outliers.

Once normalized, the per-SF scores were aggregated into a single consensus value per compound. In the MolDockLab workflow, both score-based consensus methods, like Z-Score, and rank-based consensus methods, such as RbR, and ECR method, were deployed. For each compound, only the pose with the highest consensus score was kept, and all other poses, regardless of the docking engine that produced them, were discarded.

Z-Score standardizes scores to have Z-scaled scores. This standardization, achieved by centering scores around the mean with a standard deviation of one, allows for fair comparison across different SF. This can be expressed as:

1 $${\mathrm{Z}}-{\mathrm{Score}}\,(i)=\mathop{\sum }\limits_{j=1}^{N}\frac{{f}_{ij}-{\mu }_{j}}{{\sigma }_{j}},$$

where *f*_*ij*_ is the score of compound *i* for a SF *j*,and *μ*_*j*_ and *σ*_*j*_ are the mean and standard deviation of SF *j* across all compounds.

The RbR approach assigns a rank to each pose based on individual SF, then calculates the average rank across all SF. The poses with the highest overall rankings are selected to represent each compound in the library.

Rather than averaging scores, the ECR approach combines the top-ranked molecules from each SF using an exponential distribution^80^. The resulting exponential score, *P*(*i*), is obtained by summing each molecule’s exponential rank across all SF:

2 $$P(i)=\mathop{\sum}\limits_{j}p({{r}_{i}^{j}})=\frac{1}{{\sigma }^{j}}\,\mathop{\sum}\limits_{j}\,{\exp}\,\left(-\frac{{r}_{i}^{j}}{{\sigma }^{j}}\right),$$

where ${r}_{i}^{j}$ is the rank of molecule *i* for SF *j*. The parameter *σ*^*j*^, set to the default for simplicity across all SF, *σ*^*j*^ = 0.05, serves as a weighting factor that determines the fraction of top compounds considered for each SF *j*.

Every possible combination of five docking tools, the selected SFs, and three ranking methods defines a candidate pipeline, yielding a maximum of 3,047,361 pipelines. All candidate pipelines are evaluated on three metrics: Spearman correlation coefficient (*ρ*), which measures overall ranking quality; enrichment factor (EF) at 10% (EF_10%_), which accounts for early retrieval of active compounds; and runtime cost, which captures computational expense. Formal definitions of all three metrics and hardware specifications for runtime estimation are provided in the “Performance evaluation and benchmarking”.

The pipeline with the highest *ρ* and EF_10%_ is identified, and a user-definable tolerance window is constructed by subtracting 0.1 from the maximum *ρ* and 0.5 from the maximum EF_10%_ (default values). Selection then proceeds in two stages: pipelines whose *ρ* falls within the *ρ*-tolerance form a pre-qualified pool, and the EF_10%_-tolerance is applied within that pool as a refinement. All pipelines whose *ρ* and EF_10%_ fall within this window are considered near-optimal, and the one with the lowest runtime cost among them is automatically selected and deployed for ranking larger compound sets.

### Hit selection and experimental validation

The top-ranked compounds were first triaged in silico through a three-stage post-processing funnel. Then, the selected candidates were synthesized and tested in a whole-cell uptake assay.

After computing a single consensus score for each pose, the next step is hit selection, which consists of three key stages: key interactions filtration, diversity selection, and visual inspection.

Ranked poses are analyzed to identify interactions with potential residues using the PLIP^81^. Compounds that lack key interactions in any generated pose are excluded from further consideration. Key residues are determined by profiling the interactions across all poses generated by the selected docking tools for the active compounds in the calibration set. Although the two most frequently occurring interactions are selected as key interactions by default, it is strongly recommended that users manually specify pharmacologically relevant anchor residues for filtering, as the automatic selection may yield a criteria that lack sufficient discriminative power.

From the top-ranked 1% of compounds that pass the interaction filter, a structurally diverse subset is selected. Each compound is encoded as a Morgan fingerprint (radius 2, 2048 bits) using RDKit, and K-medoids clustering is applied with the Jaccard distance metric and a user-defined number of clusters (default 10). The medoid of each cluster is returned as the representative compound, ensuring maximal chemical diversity among the final selection.

The final selection is further refined through visual inspection of the selected poses in a 3D representation within the binding pocket. This step allows to evaluate key aspects such as binding-pose stability, ligand complementarity to the binding site, potential steric clashes, and interaction quality, ensuring that the selected compounds exhibit optimal binding characteristics.

**Fig. 10:**
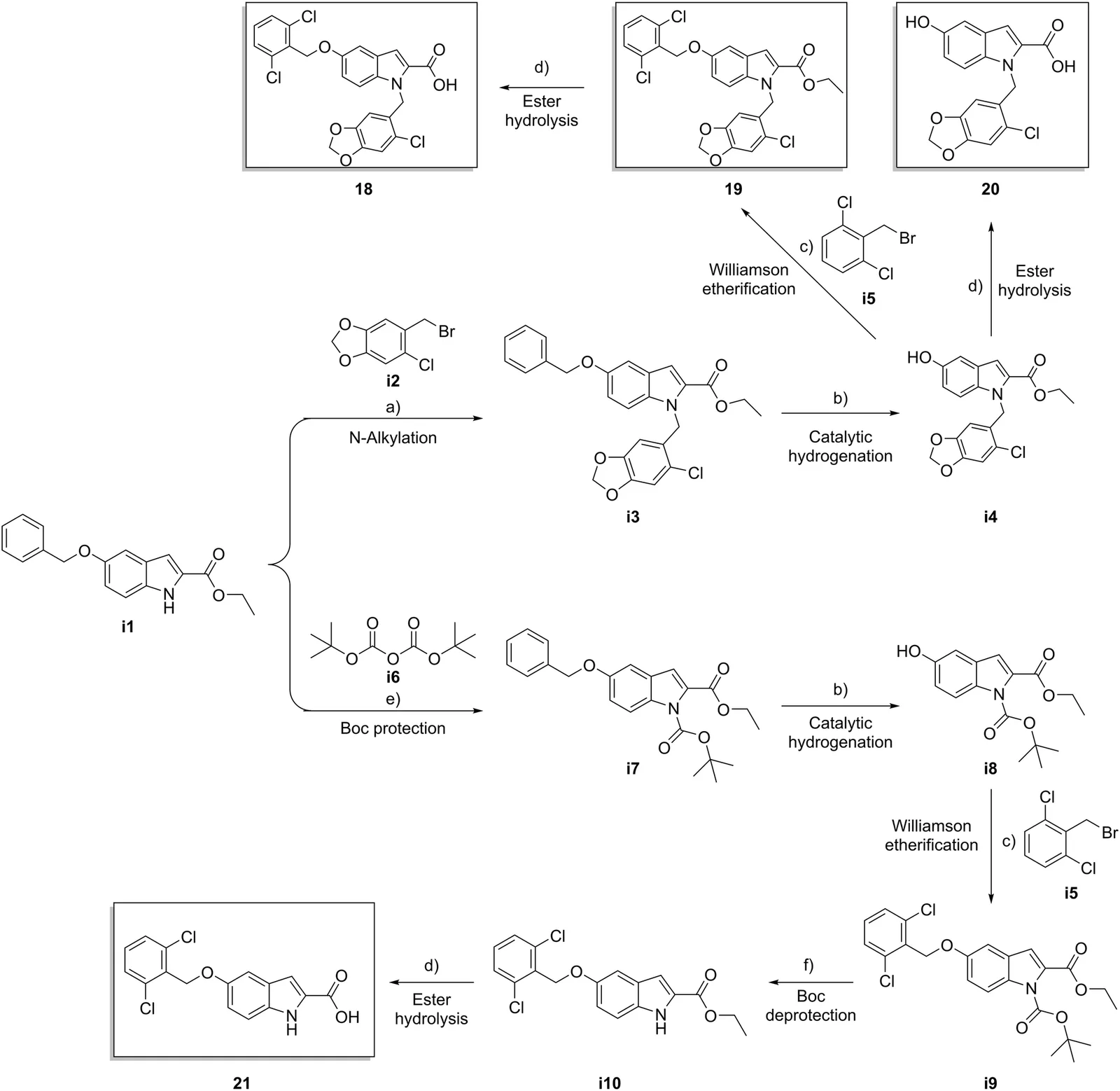
Synthetic route used to prepare compounds 18, 19, 20, and 21. Reagents and conditions: (**a**) sodium hydride, **N**, **N**-dimethylformamide, 86% yield; (**b**) 10% palladium on carbon, ethyl acetate, 18 hours, 80–89% yield; (**c**) sodium hydride, **N,N**-dimethylformamide, 76–80% yield; (**d**) tetrahydrofuran, 3 M potassium hydroxide, ethanol, reflux at 75 ℃, 71–74% yield; (**e**) 4-dimethylaminopyridine, tetrahydrofuran, room temperature, 81% yield; (**f**) trifluoroacetic acid, dichloromethane, 89% yield. Bold numbers denote the synthesized compounds.

To experimentally validate the virtual screening results, the most promising compounds emerging from the ECF-T workflow were advanced to in vitro evaluation. Herein, we outline the procedures of the synthetic schemes devised for the preparation of the potent hits, depicted in Fig. 10, followed by the whole-cell uptake assay protocol used to assess their inhibitory activity against ECF-T. Detailed general procedure (GP) for the synthesis and full spectroscopic characterization data (^1^H NMR and ^13^C NMR) for synthesized compounds are provided are given in the Supplementary Information, under General synthetic procedures“ and NMR characterization data”.

A multistep synthetic pathway was employed to synthesize the different derivatives, as outlined in Fig. 10. Initially, **N**-alkylation of **i1** was carried out using alkyl bromide **i2** in the presence of sodium hydride (NaH), affording compound **i3** in 86% yield. Compound **i2** was originally synthesized via bromination of its corresponding methyl alcohol using PBr_3_ under a nitrogen atmosphere, giving a 97% yield. Catalytic hydrogenation of **i3** using 10% palladium black removed the benzyl protecting group, yielding the phenolic derivative **i4**. This was followed by ester hydrolysis using 3 M potassium hydroxide (KOH) under reflux at 75 °C, affording compound **20** in 71% yield.

The phenolic intermediate **i4** was also coupled with 2,6-dichlorobenzyl bromide (**i5**) via Williamson etherification using NaH at room temperature, yielding compound **19**. Subsequent ester hydrolysis under the same conditions (3 M KOH, 75 °C) afforded compound **18**.

Starting again from **i1**, Boc protection was achieved using di-tert-butyl dicarbonate (**i6**) in the presence of 4-dimethylaminopyridine (DMAP), affording compound **i7** in 81% yield. Catalytic hydrogenation with 10% palladium black removed the benzyl group, yielding compound **i8**. This intermediate was subsequently coupled with **i5** via Williamson etherification, affording compound **i9** in 76% yield. Boc deprotection was carried out using trifluoroacetic acid (TFA), yielding compound **i10** in 89% yield. Finally, ester hydrolysis resulted in the free acid (**21**), in 74% yield.

The inhibitory activity of the synthesized compounds was then assessed in a whole-cell uptake assay. The ECF inhibitors and their inhibitory effect on folate uptake in the Gram-positive model organism *Lactobacillus casei* were studied in a whole-cell uptake assay as recently published in our group^68^. For this purpose, 20 *μ* L of inhibitor was added to the wells of a MultiScreenHTS Filter Plate containing 175 *μ* L of *L. casei* culture diluted in citrate buffer. The blank was determined with 185 *μ* L citrate buffer and 10 *μ* L of dimethyl sulfoxide (DMSO). 5 *μ* L of radiolabeled folic acid was added to each well (2 *μ* M, Moravek Biochemicals, Brea, CA). Using this method, we determined the percentage of inhibition at 50 *μ* M for the compounds and the IC_50_values for our best compounds. The assay was done in both technical and biological duplicates, ensuring the reliability and consistency of the data. Subsequently, the gathered data were analyzed using GraphPad Prism, a robust statistical software tool.

### Performance evaluation and benchmarking

Having defined the workflow, we describe how its outputs were evaluated: the physical validity of the generated poses, the metrics used to score and select pipelines, the robustness of the selected pipeline on unseen chemical space, and a co-folding model invoked as an external baseline.

We first assessed the physical plausibility of the generated poses. PoseBusters^32^ was invoked within MolDockLab to check the quality of the poses by enabling the –pose_quality_checker flag. For every docked pose, the tool computes 19 structural descriptors covering intrinsic ligand geometry, intramolecular steric clashes, and pose-protein interactions. Rather than discarding poses that violate one or more checks, we retained all poses and mapped the individual violations onto each pose, producing a per-pose violation profile that was used in downstream analysis to assess and compare the structural quality produced by each docking engine. The per-tool docking failure rate, defined as the fraction of ligands for which a given engine failed to produce any valid pose, was additionally recorded.

Candidate pipelines were judged on three complementary metrics, ranking quality, early enrichment, and computational cost, defined as follows: The Spearman’s correlation coefficient is a statistical measure that evaluates the strength and direction of the relationship between two variables. It uses the rank-order values, making it particularly useful when the relationship between variables is not assumed to be linear. Spearman correlation coefficient, denoted as *ρ* is calculated as

3 $$\rho =1-\frac{6\sum {d}_{i}^{2}}{n({n}^{2}-1)},$$

where *d*_*i*_ is the distance between the ranks of *i* and *n* is the number of observations.

The EF serves as a key metric for evaluating the success of virtual screening methods, quantifying the concentration of compounds classified as active within the top percentage of screened compounds. In our workflow, we have adopted the EF at a threshold of 10% due to the relatively small size of the compound list in our calibration set^82^. Stricter thresholds were considered but ultimately discarded: EF_1%_, EF_2%_ and EF_3%_ were not evaluated, since at these thresholds the top fraction of the calibration set collapses to a handful of compounds, degenerating the metric into a categorical hit/no-hit indicator rather than a continuous enrichment score. EF_5%_ was likewise excluded: with only 11 compounds in the top 5% of a 201-compound calibration set, the metric remains at the boundary of this same degeneracy regime and cannot be considered reliably resolved. EF_10%_ was therefore retained as the decisive criterion for pipeline selection. The EF at 10 is calculated by

4 $$E{F}_{10 \% }=\frac{{N}_{a}^{10 \% }}{{N}^{10 \% }}\cdot \frac{N}{{N}_{a}},$$

where ${N}_{a}^{10 \% }$ is the number of active compounds identified within the top 10% of the list, *N*^10%^ represents the total number of compounds within that top fraction, *N* is the total number of compounds screened and *N*_*a*_ denotes the overall number of active compounds.

Wall-clock runtime cost was calculated for each pipeline in the workflow. The measurements were conducted on a workstation, equipped with an Intel Xeon E3-1270 v6 CPU running at 3.80 GHz, with a maximum boost frequency of 4.20GHz. The processor features four physical cores, each supporting 2 threads, resulting in a total of 8 logical processors. The system is configured with 32 GB of RAM and utilizes an NVIDIA Quadro P1000 GPU (Pascal architecture, compute capability 6.1, 4 GB GDDR5). The operating system is Ubuntu 20.04.6 LTS. To ensure a fair runtime comparison across all docking engines and SF, every method, including DiffDock, was executed on the CPU only, with GPU acceleration disabled. For each docking engine, the mean wall-clock time from ten independent runs per ligand was recorded, while for every SF the reported runtime refers to the time needed to evaluate a single pose, computed as the average over ten independent runs, as shown in Table 7. Whereas heuristic docking methods and the deterministic FlexX algorithm produce reproducible poses, DiffDock does not, as it is a generative model that samples from a learned pose distribution. Per-ligand pipeline runtime was computed as the sum of the runtimes of all docking tools and SFs comprising the pipeline.

**Table 7:** Runtime for docking tools (per compound) and SFs (per pose) in seconds (sec)

Docking Tools	Runtime (s/cpd)
FlexX	3.33
PLANTS	6.82
SMINA	99.90
GNINA	105.80
(Local)-DiffDock	407.50
**SF**	**Runtime (s/pose)**
CHEMPLP	0.12
LinF9	0.24
AD4	0.28
Vinardo	0.29
convolutional neural network (CNN) Score	0.31
CNN Affinity	0.31
SMINA Affinity	0.31
RTMScore	0.41
RFScore V1	0.68
RFScore V2	0.69
RFScore V3	0.69
Vina Hydrophobic	0.69
Vina Intra Hydrophobic	0.69
HYDE	2.00
SCORCH	4.63

To assess the generalizability of the MolDockLab workflow across diverse datasets, we performed a 5-fold scaffold-based split evaluation on the calibration set of each case study (EGFR and ECF-T). Morgan fingerprints with a radius of 2, represented as a 2048-bit vector, based on the compounds’ Bemis-Murcko scaffolds were generated using RDKit^77^. Bemis-Murcko scaffolds, which define the core structure of a molecule, are obtained by removing peripheral side chains.

Subsequently, hierarchical density-based spatial clustering (HDBSCAN)^83^ was applied to cluster the scaffold fingerprints into groups of varying density without requiring the pre-specification of the number of clusters. Clustering was performed using the Jaccard similarity metric with a minimum cluster size of 2. This procedure yielded five distinct training-test dataset pairs, each pair underwent MolDockLab workflow separately. The performance of the workflow was assessed by comparing pipeline ranks of each train-test dataset for each fold using Pearson’s correlation. To quantify how representative each test split is of the overall chemical space of the calibration set, we report scaffold coverage, defined as the fraction of unique scaffolds in the test set to the total number of unique scaffolds in the full calibration set.

To address the growing interest in co-folding models as a complementary approach to classical docking workflows, we evaluated the open-source model Boltz-2^29^ on the EGFR calibration set as an additional baseline. Boltz-2 was used through its command-line interface with the recommended set-up for affinity-aware predictions: three recycling steps, 100 sampling steps, ten diffusion samples per ligand, and the inference-time physicality steering potentials enabled (--recycling_steps 3 --sampling_steps 100 --diffusion_samples 10 --use_potentials).

For each calibration compound, the model was provided with a structured YAML input specifying the protein sequence, the ligand SMILES, a binding-pocket constraint, and a structural template. The protein sequence corresponded to the EGFR kinase domain was retrieved from UniProt (entry P00533), and the ligand was supplied as a canonical SMILES string generated from the prepared 3D conformer. To restrict the predicted complex to the same binding site used by the classical docking engines, a pocket constraint was defined listing the residues lining the ATP-binding cleft of the prepared 5UG9 structure (those within 5 Å of the co-crystallized ligand), with a maximum binder–residue distance of 6 Å and the constraint flagged as enforced (force: true). In addition, the prepared apo structure of 5UG9 was supplied as an enforced structural template (force: true) with an RMSD threshold of 1.5 Å, ensuring that the predicted protein conformation remained consistent with the receptor used by all other pipelines in this study. The highest-confidence pose returned by Boltz-2 was retained for scoring, and the model’s predicted binding affinity was used as the ranking signal.

In contrast to all other methods reported in this study, the Boltz-2 baseline could not be executed within practical wall-time on the CPU-only workstation described before, and was therefore run on a separate compute node. This node is equipped with two AMD EPYC 7662 64-core processors (128 physical cores, 256 logical threads in total) arranged across two NUMA domains, 1 TiB of DDR4 system memory, one NVIDIA A100-PCIE 40 GB GPU, and local NVMe storage. A CPU-only Boltz-2 configuration on the same node also failed to complete a single ligand within practical wall-time and is therefore not reported.

On this hardware, Boltz-2 required 203.4 ± 1.0 s per compound (mean ± standard deviation, *n* = 10). We emphasize that the Boltz-2 runtime is not directly comparable to the figures reported in Table 7, as it was obtained on different hardware and with GPU acceleration; it is provided as a stand-alone reference for the order-of-magnitude cost of co-folding at screening scale.

## Supplementary information

## Data Availability

The data supporting the findings of this study are available on Zenodo at https://zenodo.org/records/19384927.

## Code availability

The source code for MolDockLab is publicly available on GitHub at https://github.com/volkamerlab/MolDockLab/. FlexX is a commercial product distributed by BioSolveIT GmbH and requires a valid license, whereas the remaining four docking engines are openly available for academic use. Likewise, SF HYDE is a commercial product distributed by BioSolveIT GmbH and requires a valid license, while the remaining SFs are openly available. Note that MolDockLab can be used without integration of commercial software, and users without FlexX and HYDE licenses can run the workflow with the open-source docking engines and SFs.
