# Supplementary material for: Data driven selection of consensus docking pipelines for structure based hit identification
Source: NPJ Drug Discov. 2026 Sep 2;3:37. doi: 10.1038/s44386-026-00063-4 (PMC13538370; doi:10.1038/s44386-026-00063-4)
Supplement: Supplementary file 1 [file 44386_2026_63_MOESM1_ESM.pdf]

Supplementary Information

1 Activity threshold analysis of EGFR

To demonstrate that this choice does not unduly drive the pipeline selection, we performed a sensitivity analysis across activity thresholds spanning $\mathrm{pIC}_{50} \geq 6.0$ to $\mathrm{pIC}_{50} \geq 9.0$, in steps of 0.5.

For each threshold, we recomputed the per-pipeline enrichment factor (EF) on the calibration set, yielding one EF vector per cutoff. Pairwise Spearman rank correlations between these vectors then quantify the agreement between pipeline rankings obtained at different cutoffs: high correlations indicate that the relative ordering of pipelines is consistent across thresholds, whereas low correlations indicate that the choice of cutoff substantially alters which pipelines appear best (Supplementary Figure 1).

Pipeline ordering is highly preserved between adjacent cutoffs in the meaningful range, with $\rho = 0.93$ between $\mathrm{pIC}_{50} \geq 6.5$ and $\mathrm{pIC}_{50} \geq 7.0$, and $\rho = 0.85$ between $\mathrm{pIC}_{50} \geq 7.5$ and $\mathrm{pIC}_{50} \geq 8.0$. Correlation degrades substantially starting from $\mathrm{pIC}_{50} \geq 8.5$ (with all pairwise $\rho \lesssim 0.21$), where the active set becomes too small to discriminate pipelines and the EF metric takes only a few discrete values.

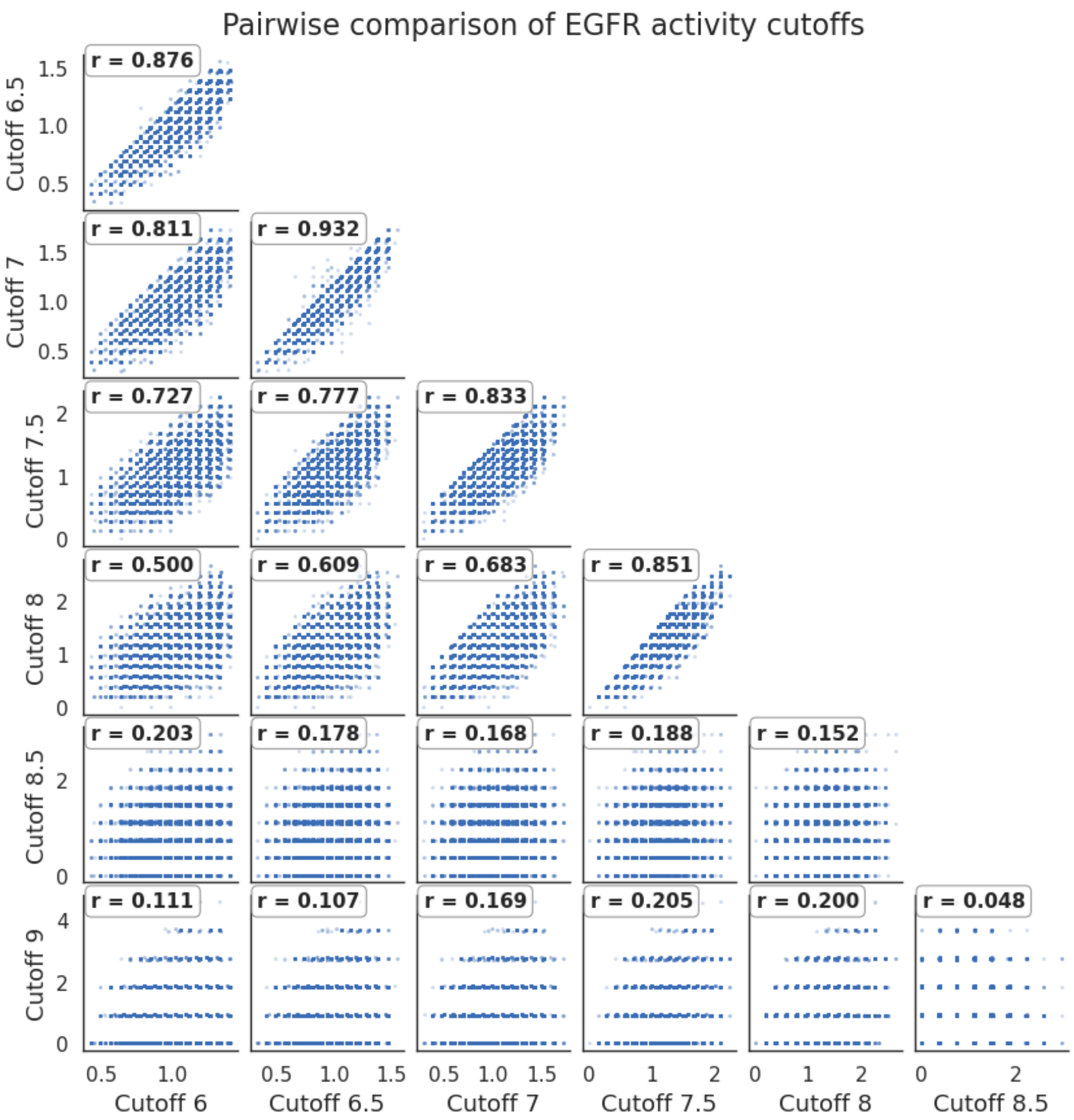

Supplementary Figure 1. Sensitivity of per-pipeline enrichment factors to the activity threshold used to define active compounds for the epidermal growth factor receptor. Pairwise comparison of per-pipeline enrichment factors computed at different pIC50 (the negative base-10 logarithm of the half-maximal inhibitory concentration) activity thresholds for the epidermal growth factor receptor. In each off-diagonal panel, every point represents one pipeline and the two axes give its enrichment factor at the two thresholds being compared; the annotation gives the Spearman rank correlation between the two enrichment-factor vectors. The diagonal panels show the distribution of enrichment-factor values at each threshold. Pipeline ordering is well preserved between adjacent thresholds in the meaningful range and degrades only at a pIC50 of 9.0 or above, where the active set becomes too small to discriminate pipelines.

2 Hodge ranking validation

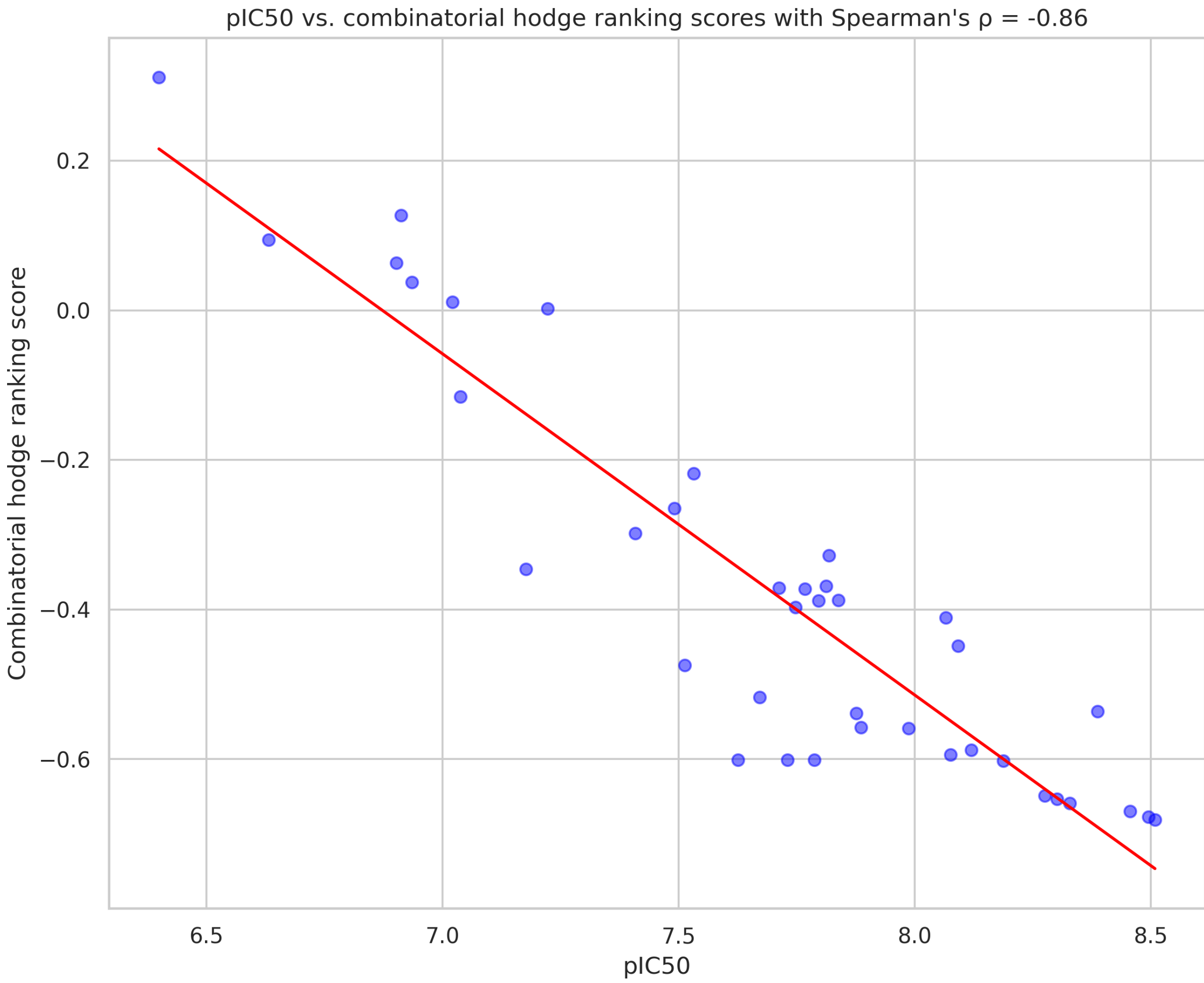

Supplementary Figure 2. Agreement between Hodge-ranking scores and measured potencies for the validated ECF-T compounds. Relationship between the combinatorial Hodge-ranking score and the pIC50 (the negative base-10 logarithm of the half-maximal inhibitory concentration) of the 38 compounds with experimentally measured potencies in the energy-coupling factor transporter (ECF-T) dataset. The strong negative correlation indicates that the Hodge-ranking scheme is predictive of measured potency.

3 Technical details of scoring functions

The specific implementations, versions, and input requirements of each SF are detailed below and summarized in Table 1. Affinity predictions from SMINA, AD4, Vinardo, and convolutional neural network (CNN)-based methods were performed with `gnina` v1.0 using a fixed seed for reproducibility.

The RF-Score suite (V1, V2, and V3), as well as Vina's hydrophobic and intra-hydrophobic scoring functions (SFs), were implemented using the `ODDT` 0.8 python package [?].

LinF9, CHEMPLP, and HYDE were run using their respective executables: `smina.static`, `plants` (v1.0 with `--mode rescore`), and `hydescorer` v2.0. CHEMPLP determined the coordinates directly using its own executable, while HYDE and LinF9 employed a reference ligand. This reference ligand was provided in SDF format for HYDE and protein data bank (PDB) format for LinF9. HYDE produced a range of potential scores, from which the lower boundary was selected as the final affinity value.

SCORCH and RTMScore were accessed from their respective repositories and employed with default parameters. RTMScore used the binding pocket as input rather than the entire protein structure. Among the three RTMScore models (trained with different seeds), the first was chosen arbitrarily for evaluation. The category and executable used for each SF are summarised in Table 1.

Supplementary Table 1. SFs categories and the executable files used.

**Scoring Function**	**SF type**	**Executable File**
SMINA affinity	Empirical	gnina v1.0
AD4	Empirical	gnina v1.0
Vinardo	Empirical	gnina v1.0
Vina Hydrophobic	Empirical	ODDT package
Intra-Hydrophobic	Empirical	ODDT package
LinF9	Empirical	smina.static
ChemPLP	Empirical	plants v1.0
HYDE	Empirical	hydescorer v2.0
SCORCH	ML-based	SCORCH github
RTMScore	ML-based	RTMScore github
CNN affinity/Score	ML-based	gnina v1.0
RF-Score V1	ML-based	ODDT package
RF-Score V2	ML-based	ODDT package
RF-Score V3	ML-based	ODDT package

4 Normalized SF score distributions

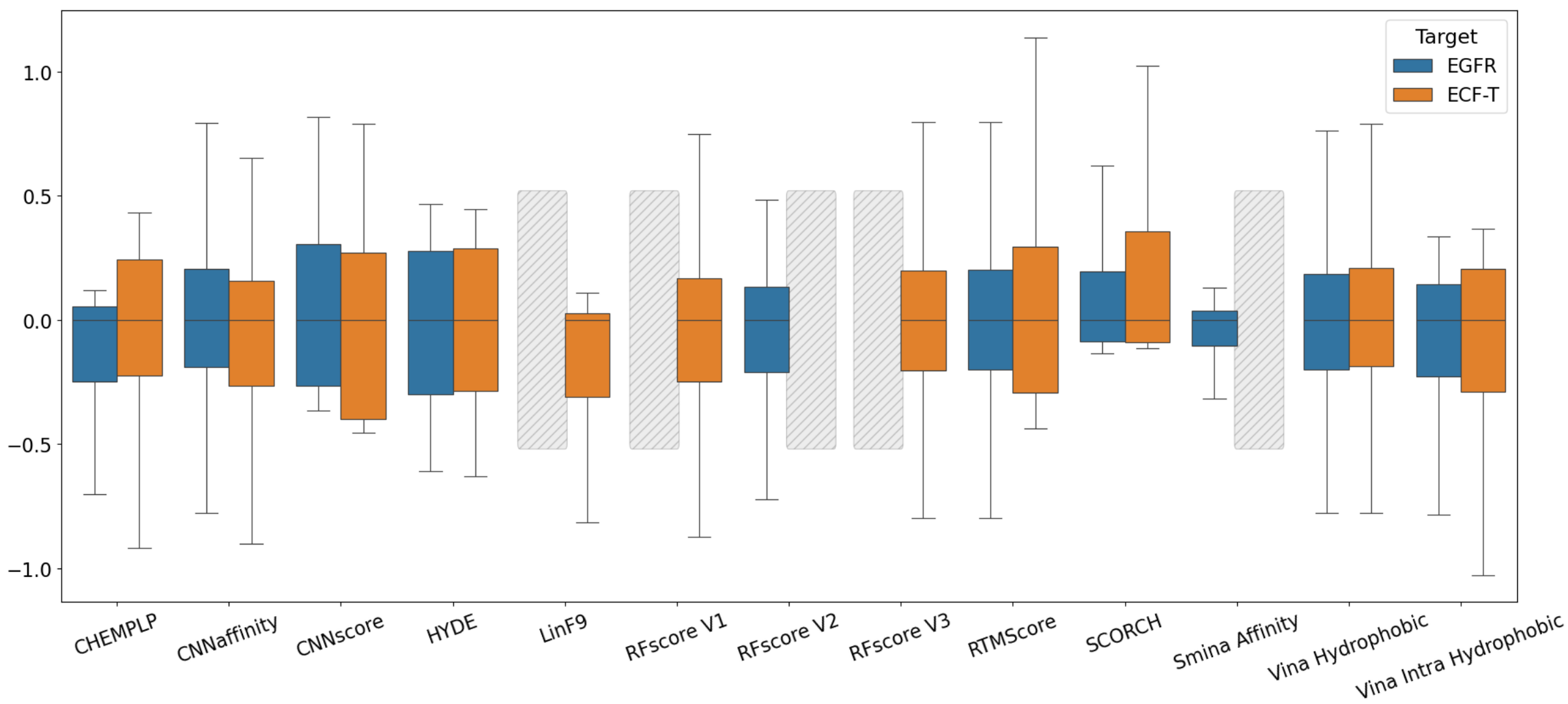

Supplementary Figure 3. Normalised score distributions of all scoring functions for both targets. Normalised predicted-score distributions for each scoring function, shown for the epidermal growth factor receptor (EGFR, blue) and the energy-coupling factor transporter (ECF-T, orange). Each box shows the median and interquartile range for one scoring function; outliers are omitted for clarity. Scoring functions are listed along the horizontal axis and normalised scores on the vertical axis. Hatched grey boxes mark scoring functions excluded for a given target by the redundancy filter. SF, scoring function.

5 Pairwise correlation matrices of SFs

We computed pairwise Spearman's correlations for each pairs of SFs to identify SFs that behave similarly. The resulting correlation matrices are shown for epidermal growth factor receptor (EGFR) in Supplementary Figure 4 and for energy coupling factor transporters (ECF-T) in Supplementary Figure 5. SMINA affinity, AD4, LinF9, and Vinardo exhibited highly positive mutual correlations, all exceeding 0.9. Similarly, RFScore V1, RFScore V2, and RFScore V3 were also found to be strongly correlated with each other.

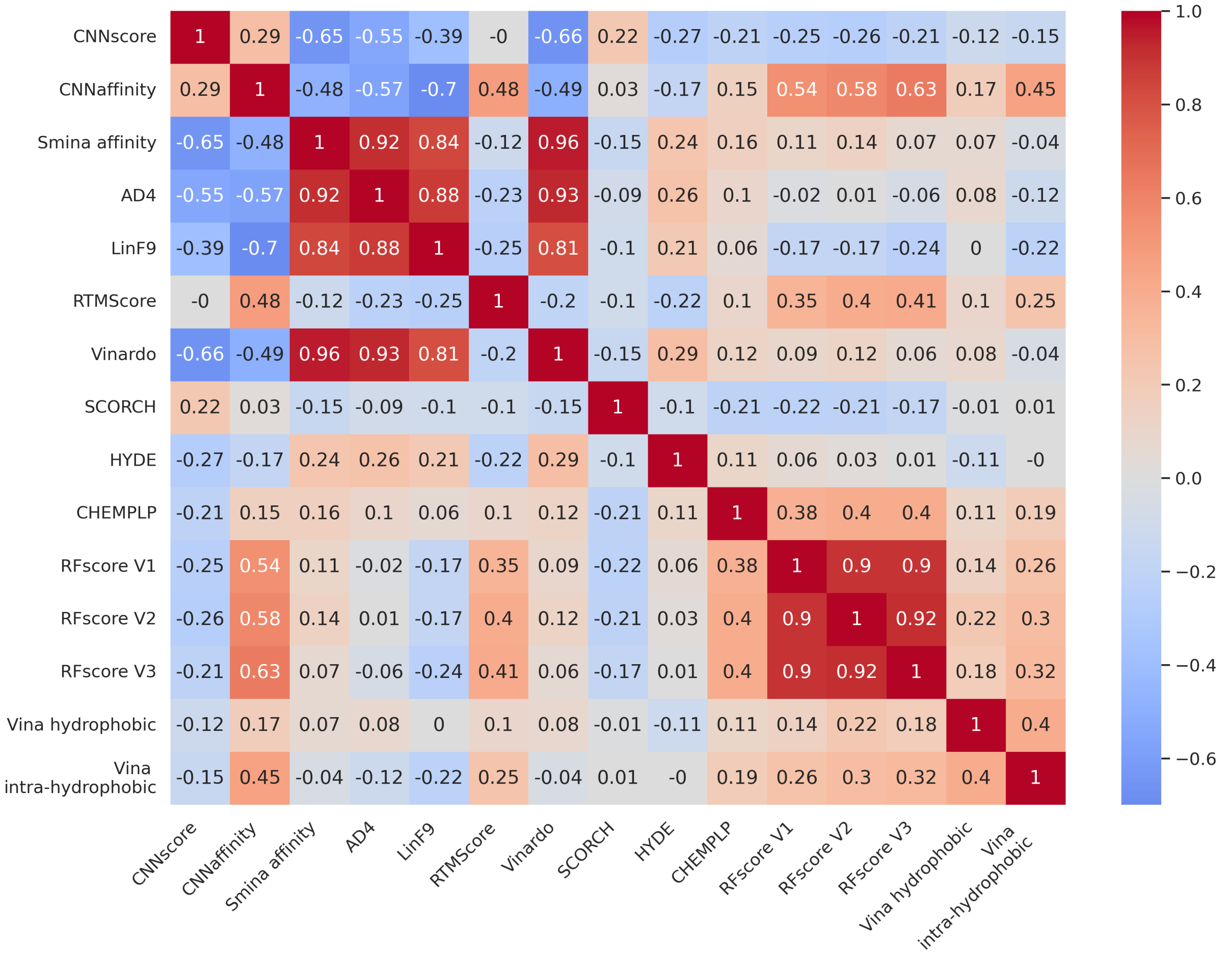

Supplementary Figure 4. Pairwise correlation between scoring functions for the epidermal growth factor receptor target. Matrix of Spearman correlation coefficients between all scoring functions evaluated on the epidermal growth factor receptor (EGFR) target. Cell shading encodes the strength of correlation, with darker cells indicating stronger positive correlation between the two scoring functions.

Whereas for ECF-T target, SMINA affinity, AD4, LinF9, and Vinardo SFs demonstrated identical ranking orders provided by these SFs with a Spearman correlation of 1.0. Similarly, RFScore V2 and V3 were closely correlated with a Spearman correlation 0.93.

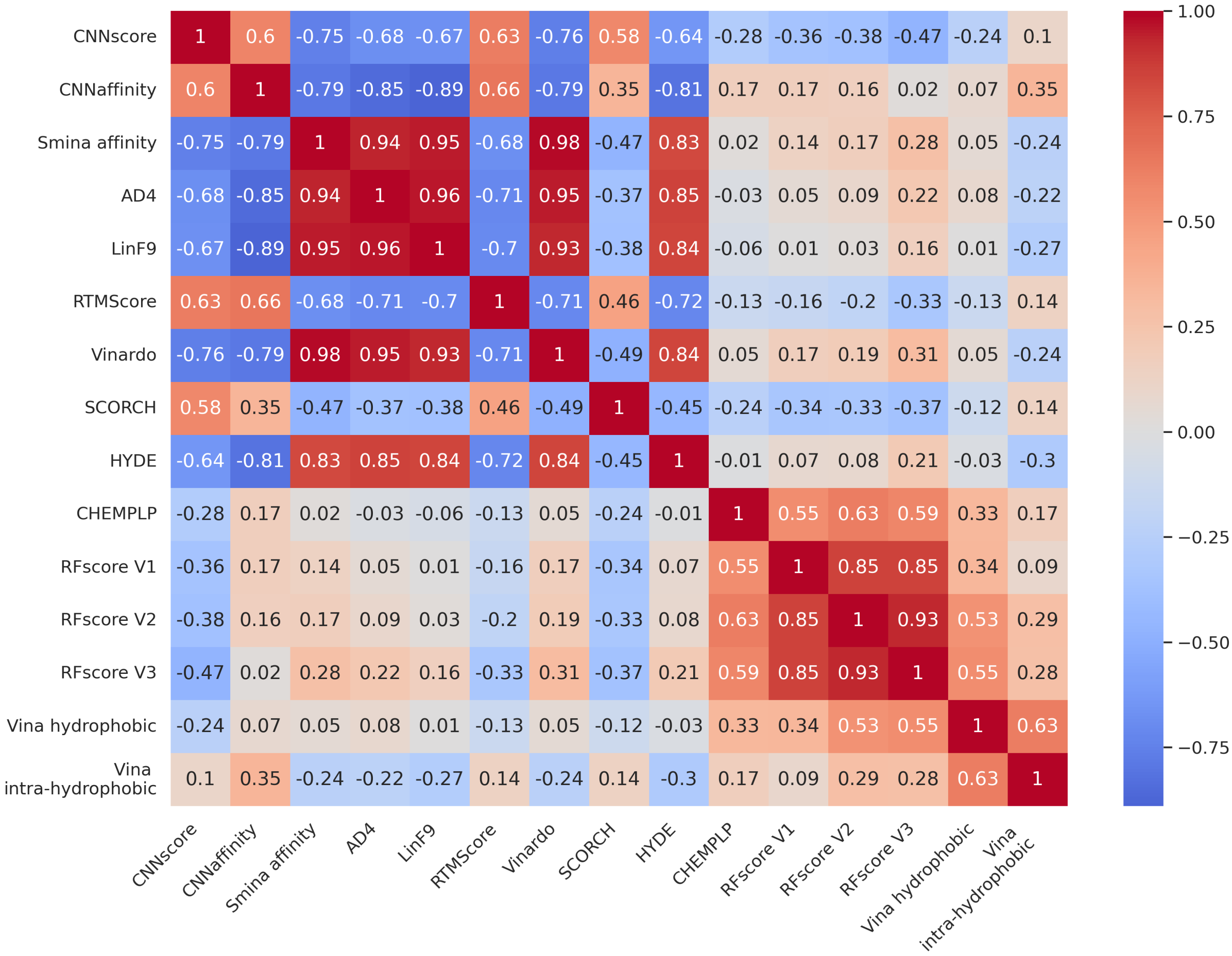

Supplementary Figure 5. Pairwise correlation between scoring functions for the energy-coupling factor transporter target. Matrix of Spearman correlation coefficients between all scoring functions evaluated on the energy-coupling factor transporter (ECF-T) target. Cell shading encodes the strength of correlation, with darker cells indicating stronger positive correlation between the two scoring functions.

6 Detailed PoseBusters validation results

6.1 Chemical and intramolecular validation

For the EGFR target, all poses generated with the diverse docking tools passed the RDKit-based chemical validity checks; however, DiffDock produced 6 poses that did not satisfy double-bond stereochemistry requirements. For the ECF-T target, all generated poses across all methods met the chemical validity criteria. Regarding intramolecular validation for EGFR, internal steric clashes were detected to varying extents across tools (Supplementary Figure 6): DiffDock showed the highest number with 85 affected poses, followed by PLANTS with 56, GNINA with 26, SMINA with 15, and FlexX with 13 poses. Increased conformational energies were observed in 144 SMINA poses and 138 GNINA poses, compared to 115 for DiffDock, 111 for PLANTS, and 92 for FlexX. For the ECF-T target, intramolecular issues were overall less frequent. PLANTS exhibited the highest number of clashes (49 poses), followed by localDiffDock with 12, SMINA with 15, and GNINA with 3 poses, whereas FlexX produced no steric clashes. Elevated conformational energies were also reduced compared to EGFR, with 48 SMINA poses, 46 PLANTS poses, 43 GNINA poses, 25 localDiffDock poses, and 2 FlexX poses displaying higher internal strain. Together, these results indicate that while all tools maintain baseline chemical validity, their ability to control intramolecular strain varies considerably, with FlexX

consistently producing the fewest issues across both targets.

6.2 Intermolecular validation

As shown in Supplementary Figure 6, intermolecular validation revealed substantial differences among docking tools. For the EGFR target, DiffDock exhibited the weakest performance, with 92 % of its poses violating minimum protein–ligand distance criteria and 1734 poses showing volume overlap with the receptor. FlexX and PLANTS showed moderate rates of minimum-distance violations (702 and 633 poses, respectively); notably, FlexX generated no poses with volume overlap with the protein, while PLANTS produced 52 such poses. SMINA and GNINA maintained high intermolecular accuracy, with only 10 and 1 violations, respectively. For the ECF-T target, localDiffDock failed entirely in intermolecular validation, with all poses violating the minimum-distance threshold and showing volume overlaps. FlexX and PLANTS performed more reliably, with violation rates of 8.2 % and 4.16 %, respectively, accompanied by only minimal overlap events. As observed for EGFR, SMINA and GNINA again achieved near-perfect adherence to intermolecular criteria.

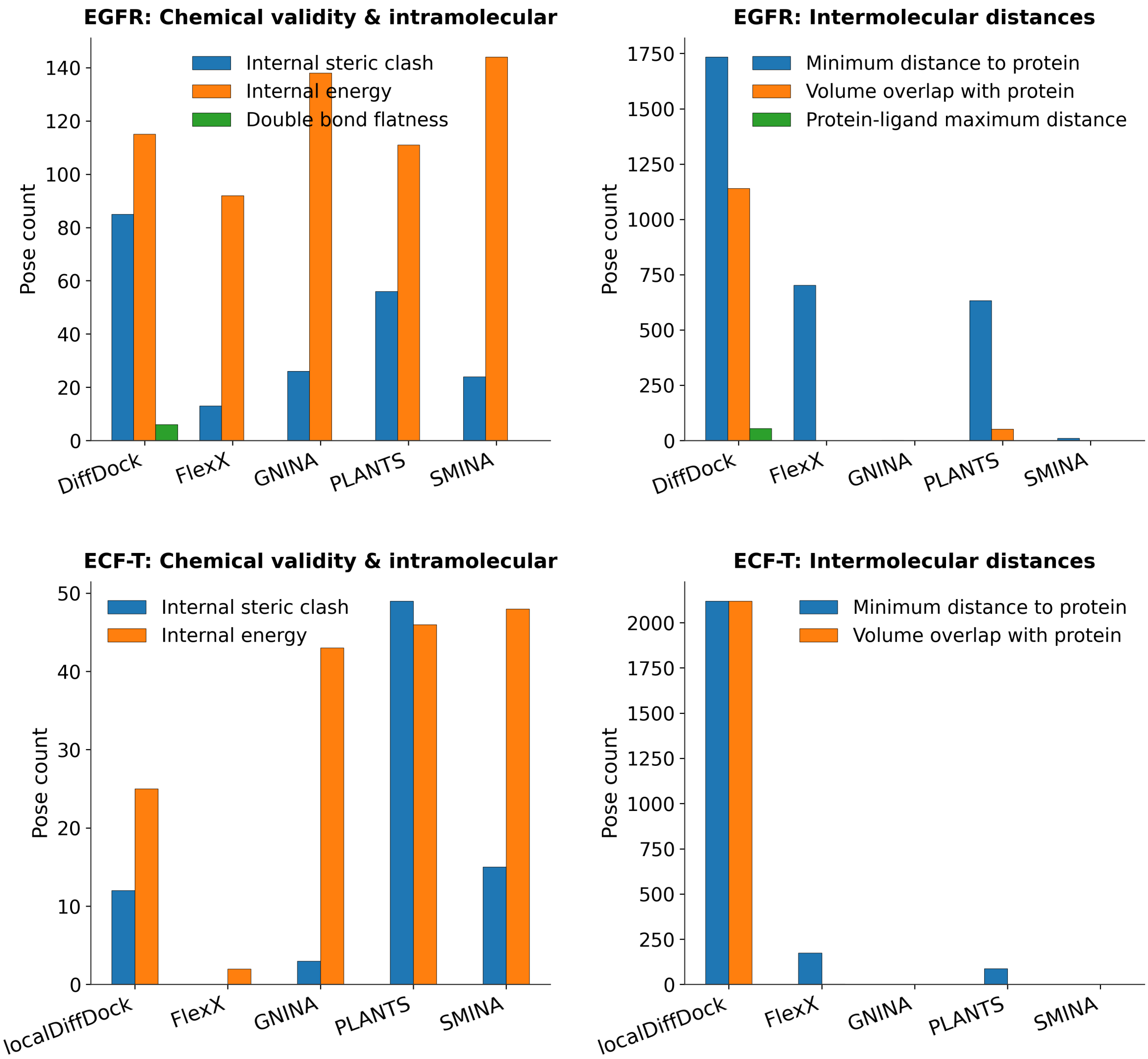

Supplementary Figure 6. PoseBusters quality assessment of poses from the five docking methods for both targets. Docking-quality comparison of the five methods (local DiffDock, FlexX, GNINA, PLANTS, and SMINA) assessed with PoseBusters for the epidermal growth factor receptor (EGFR, top row) and the energy-coupling factor transporter (ECF-T, bottom row). The left panels report chemical validity and intramolecular strain (internal steric clashes, internal energy, and bond flatness); the right panels report intermolecular quality (minimum protein–ligand distance, volume overlap with the protein, and maximum protein–ligand distance).

7 Per-tool baselines on EGFR

The naïve baseline reported in Table 4 is the mean across all five docking engines used in this work, each using its own native scoring function with no rescoring and no consensus. The per-tool breakdown for the calibration and screening sets is provided in Supplementary Table 1 and Supplementary Table 2, respectively.

Supplementary Table 2. Per-tool single-engine baseline on the EGFR calibration set ($N = 201$). $EF_{1\%}$, $EF_{3\%}$, and $EF_{5\%}$ are not reported for DiffDock as these were not part of the original calibration analysis for this tool.

Tool	Native SF	n_{cpd}	ρ	$EF_{5\%}$	$EF_{10\%}$
PLANTS	CHEMPLP	193	0.0027	–	0.813
GNINA	CNNaffinity	201	0.2904	–	1.577
SMINA	minimizedAffinity	201	-0.0808	–	0.788
FlexX	BIOSOLVEIT.DOCKING_SCORE	199	-0.0337	–	0.976
DiffDock	confidence score	188	-0.040	–	0.970
**Mean ± SD**			0.027 ± 0.147	1.17 ± 0.46	1.03 ± 0.32

Supplementary Table 3. Per-tool single-engine baseline on the EGFR screening set.

Tool	Native SF	n_{cpd}	ρ	$EF_{1\%}$	$EF_{3\%}$	$EF_{5\%}$	$EF_{10\%}$
GNINA	CNNaffinity	6458	0.303	1.004	0.988	0.994	0.997
FlexX	BIOSOLVEIT.DOCKING_SCORE	6300	0.012	1.004	1.004	1.004	0.999
SMINA	minimizedAffinity	6458	-0.051	1.004	1.004	1.001	0.999
PLANTS	CHEMPLP	6180	-0.085	1.004	1.004	0.997	1.001
DiffDock	confidence_score	5991	-0.135	0.391	0.456	0.613	0.692
**Mean ± SD**			0.009 ± 0.173	0.88 ± 0.27	0.89 ± 0.24	0.92 ± 0.17	0.94 ± 0.14

8 Distribution of pIC_{50} after PLIP filtering

The effect of the protein–ligand interaction profiler (PLIP)-based anchor-residue filter on the activity distribution of the EGFR screening set is illustrated in Supplementary Figure 7. The filter retains nearly the entire active population while only modestly depleting the inactive tail, consistent with the high sensitivity (99.2 %) and low specificity (3.5 %) reported in the main text.

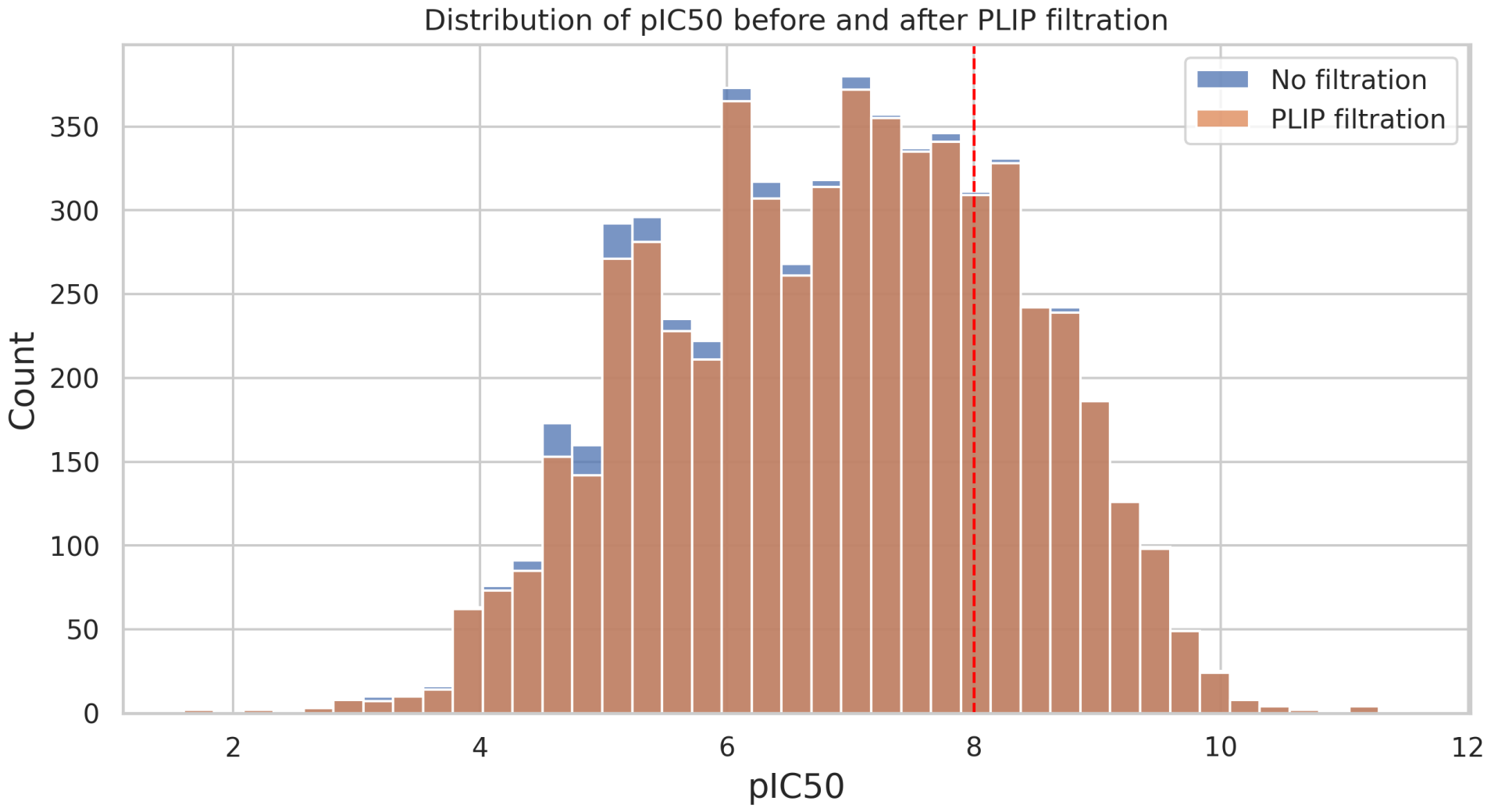

Supplementary Figure 7. Effect of the interaction-based filter on the activity distribution of the EGFR screening set. Distribution of pIC50 (the negative base-10 logarithm of the half-maximal inhibitory concentration) values for the complete epidermal growth factor receptor (EGFR) screening set before filtering (no filtration) and after filtering by the key protein–ligand contacts detected with the Protein–Ligand Interaction Profiler (methionine 793 and phenylalanine 856). The red dashed vertical line at a pIC50 of 8 marks the threshold used to label compounds as active.

General synthetic procedures

[id=new]The general procedures referenced above are as follows. General procedure A (GP-A), N-alkylation: A solution of sodium hydride (NaH) (60 % dispersion in mineral oil, 1.5 eq.) in N,N-Dimethylformamide (DMF), ethyl 5-(benzyloxy)-1H-indole-2-carboxylate (1 eq.) was added. The resulting mixture was stirred at room temperature for 60 min. After that, 6-Chloropiperonyl bromide (1 eq.) was added to the reaction mixture. The resulting mixture was stirred at room temperature for 5 h. The resulting mixture was poured into ice water and extracted using ethyl acetate (EtOAc). The combined organic layer was washed with cold H_2O and cold brine, dried using magnesium sulfate ($MgSO_4$), filtered, concentrated in vacuo, and purified by flash chromatography.

General procedure B (GP-B), catalytic hydrogenation: To a solution of the benzyl-substituted ester (1 eq.) in EtOAc, 10 % Pd on carbon (0.1 eq.) was added. The mixture was stirred under hydrogen using a Double-walled balloon for 18 h. The reaction was monitored using LC-MS. After complete conversion, the afforded mixture was filtered over Celite, concentrated in vacuo, and purified by flash chromatography.

General procedure C (GP-C), Williamson etherification: To a solution of NaH (60 % dispersion in mineral oil, 1.5 eq.) in DMF, the corresponding phenol derivative (1.0 eq.) was added. The reaction mixture was stirred at room temperature for 1 h, then the following 2,6-dichlorobenzyl bromide (1.12 eq.) was added. The reaction mixture was stirred at room temperature for an additional hour. After that, the resulting mixture was poured into ice water and extracted using EtOAc (3x). The combined organic layer was washed with cold H_2O, cold brine, dried using $MgSO_4$, filtered, concentrated in vacuo, and purified by flash chromatography.

General procedure D (GP-D), ester hydrolysis: The corresponding ester (1.0 eq.) was dissolved in tetrahydrofuran (THF), ethanol (EtOH), and 3M potassium hydroxide (KOH) (1:1:1 ratio). The resulting mixture was refluxed for 2 h at 75°C. The reaction was monitored using LC-MS. After complete conversion, the EtOH was removed under vacuum, and the pH of the

aqueous phase was initially adjusted to 3 with 3M HCl and then extracted with EtOAc (3x). Finally, the combined organic layer was partitioned between EtOAc and 3M HCl, and it was washed with H_2O and brine, dried using $MgSO_4$, filtered, and concentrated in vacuo. The crude was purified via preparative HPLC.

General procedure E (GP-E), Boc protection: A solution of the corresponding indole derivative (1.0 eq.) was dissolved in THF, and 4-DMAP (0.2 eq.) and BOC_2O (1.2 eq.) were added. The reaction mixture was stirred at rt until the amine was converted entirely. Afterwards, the resulting mixture was extracted with EtOAc (3x) and concentrated in vacuo.

General procedure F (GP-F), Boc deprotection: To a solution of the N-Boc-protected indole derivative (1.0 eq) in dichloromethane (DCM), trifluoroacetic acid (TFA) (1.0 ml) is added dropwise at room temperature. The resulting mixture is stirred at room temperature for approximately 2 h, and the reaction progress was monitored via LC-MS. Upon completion, the reaction mixture was diluted with additional DCM and washed sequentially with 1M aqueous sodium bicarbonate ($NaHCO_3$) (3x) and brine to neutralize and remove excess TFA. The organic phase was dried using $MgSO_4$, filtered, and concentrated in vacuo.

9 NMR characterisation data

Compound 18:

1-((6-Chlorobenzo[d][1,3]dioxol-5-yl)methyl)-5-((2,6-dichlorobenzyl)oxy)-1H-indole-2-carboxylic acid

According to **GP-D**, compound **18** was formed using **19** (31.0 mg, 62.20 µmol), KOH (1.3 ml), EtOH (1.3 ml), and THF (1.3 ml). The resulting material was purified by preparative HPLC, and compound **18** was isolated as a white solid (27.0 mg, 71 %).

^{1}H NMR (500 MHz, DMSO-d_6): δ = 5.25 (s, 2H), 5.56 (s, 1H), 5.79 (s, 2H), 5.96 (s, 2H), 7.01 (dd, ^{3}J = 9.1 Hz, ^{4}J = 2.1 Hz, 1H), 7.13 (s, 1H), 7.30 (s, 1H), 7.32 (d, ^{3}J = 9.1 Hz, 1H), 7.42 (d, ^{4}J = 2.1 Hz, 1H), 7.46 (t, ^{3}J = 8.2 Hz, 1H), 7.56 (d, ^{3}J = 8.2 Hz, 2H), 12.98 (bs, 1H).

^{13}C-NMR (125 MHz, DMSO-d_6): δ = 45.1, 65.4, 102.0, 104.3, 105.7, 109.7, 110.2, 111.9, 116.8, 122.4, 125.9, 128.66, 128.73, 129.2, 131.5, 131.9, 134.54, 136.1, 146.9, 147.0, 153.6, 162.6.

HRMS (ESI): calcd for $C_{24}H_{16}Cl_3NO_5$ $[M–H]^-$: 502.0021, found 502.0028.

Compound 19:

Ethyl 1-((6-chlorobenzo[d][1,3]dioxol-5-yl)methyl)-5-((2,6-dichlorobenzyl)oxy)-1H-indole-2-carboxylate

According to **GP-C**, compound **19** was synthesized using **i4** (50.0 mg, 133.77 µmol), NaH (8.0 mg, 200.65 µmol), and **i5** (36.0 mg, 149.82 µmol). The resulting crude was purified using flash chromatography (c-Hex/EtOAc 8:2). The desired compound was isolated as a white solid (46.0 mg, 65 %).

^{1}H NMR (500 MHz, $CDCl_3$): δ = 1.29 (t, ^{3}J = 7.3 Hz, 3H), 4.26 (q, ^{3}J = 7.3 Hz, 2H), 5.24 (s, 2H), 5.70 (s, 2H), 5.73 (s, 1H), 5.79 (s, 2H), 6.80 (s, 1H), 6.99 (dd, ^{3}J = 9.1 Hz, ^{4}J = 2.4 Hz, 1H), 7.09 (d, ^{3}J = 9.1 Hz, 1H), 7.16 (d, ^{3}J = 8.5 Hz, 1H), 7.23 (d, ^{4}J = 2.4 Hz, 1H), 7.29–7.31 (m, 3H).

^{13}C-NMR (125 MHz, $CDCl_3$): δ = 14.3, 45.8, 60.7, 66.0, 101.6, 104.8, 106.7, 109.7, 110.7, 111.6, 117.7, 123.3, 126.4, 128.2, 128.5, 129.2, 130.3, 132.4, 135.2, 137.1, 147.1, 154.2, 161.8.

LC-MS calcd for $C_{26}H_{20}Cl_3NO_5$ $[M+H]^+$: 532.0, found: 532.0.

Compound 20:

1-((6-Chlorobenzo[d][1,3]dioxol-5-yl)methyl)-5-hydroxy-1H-indole-2-carboxylic acid

According to **GP-D**, compound **20** was formed using **i4** (50.00 mg, 133.77 µmol), KOH (1.6 ml), EtOH (1.6 ml), and THF (1.6 ml). The resulting material was purified by preparative HPLC, and compound **20** was isolated as a white solid (33.0 mg, 71 %).

^{1}H NMR (500 MHz, DMSO-d$_6$): δ = 5.52 (s, 1H), 5.74 (s, 2H), 5.97 (s, 2H), 6.85 (dd, J = 8.85, 2.29 Hz, 1H), 7.01 (d, J = 2.29 Hz, 1H), 7.15 (s, 1H), 7.19–7.20 (m, 1H), 7.21 (d, J = 9.00 Hz, 1H), 9.09 (s, 1H), 12.88 (br s, 1H).

^{13}C NMR (DMSO-d$_6$, 126 MHz): δ = 45.4, 102.5, 105.6, 106.1, 110.1, 110.2, 111.9, 117.0, 122.8, 126.8, 128.7, 129.9, 134.2, 147.3, 147.4, 152.5, 163.2.

HRMS (ESI): calcd for $C_{17}H_{12}ClNO_5$ [M–H]$^-$: 344.0331, found 344.0335.

Compound 21:
5-((2,6-Dichlorobenzyl)oxy)-1H-indole-2-carboxylic acid

According to **GP-D**, compound **21** was formed using **i10** (80.0 mg, 219.65 µmol), KOH (2.0 ml), EtOH (2.0 ml), and THF (2.0 ml). The resulting material was purified by preparative HPLC, and compound **21** was isolated as a white solid (55.0 mg, 74 %).

^{1}H NMR (500 MHz, DMSO-d$_6$): δ = 5.24 (s, 2H), 6.96 (dd, J = 9.00, 2.44 Hz, 1H), 7.03 (dd, J = 2.14, 0.76 Hz, 1H), 7.33 (d, J = 2.44 Hz, 1H), 7.36 (d, J = 9.00 Hz, 1H), 7.45–7.51 (m, 1H), 7.56–7.59 (m, 2H), 11.67 (s, 1H), 12.90 (br s, 1H).

^{13}C NMR (DMSO-d$_6$, 126 MHz): δ = 65.9, 104.3, 107.4, 113.9, 116.6, 127.6, 129.2, 129.4, 131.9, 132.6, 133.4, 136.6, 153.5, 163.2.

HRMS (ESI): calcd for $C_{16}H_{11}Cl_2NO_3$ [M–H]$^-$: 334.0043, found 334.0047.

10 Pipelines within tolerance range

Supplementary Table 4. Pipelines for EGFR ranked by Spearman correlation within tolerance ranges of Spearman correlation and $EF_{10\,\%}$. A ranking method is omitted when only a single SF is used.

Docking tool(s)	SFs	Ranking method	ρ	$EF_{10\,\%}$	Runtime cost
DiffDock	CHEMPLP, RTMScore	Z-score	0.4604	1.94	412.81
DiffDock	RTMScore	–	0.4602	1.94	411.60
DiffDock, SMINA	RTMScore, SCORCH	Rank by Rank	0.460	2.09	557.80
DiffDock, GNINA, SMINA	RTMScore, SCORCH	Rank by Rank	0.453	2.09	663.60

Supplementary Table 5. Pipelines for ECF-T ranked by Spearman correlation within tolerance ranges of Spearman correlation and $EF_{10\,\%}$.

Docking tool(s)	SFs	Ranking method	ρ	$EF_{10\,\%}$	Runtime cost
PLANTS	CNNaffinity, SCORCH, RFScore V3	Rank by Rank	0.448	2.95	63.12
PLANTS	CNNaffinity, SCORCH, RFScore V3	Z-score	0.447	3.13	63.12
PLANTS, FlexX	CNNaffinity, SCORCH, RFScore V3	Rank by Rank	0.445	2.94	66.45

11 ECF-T selected compounds

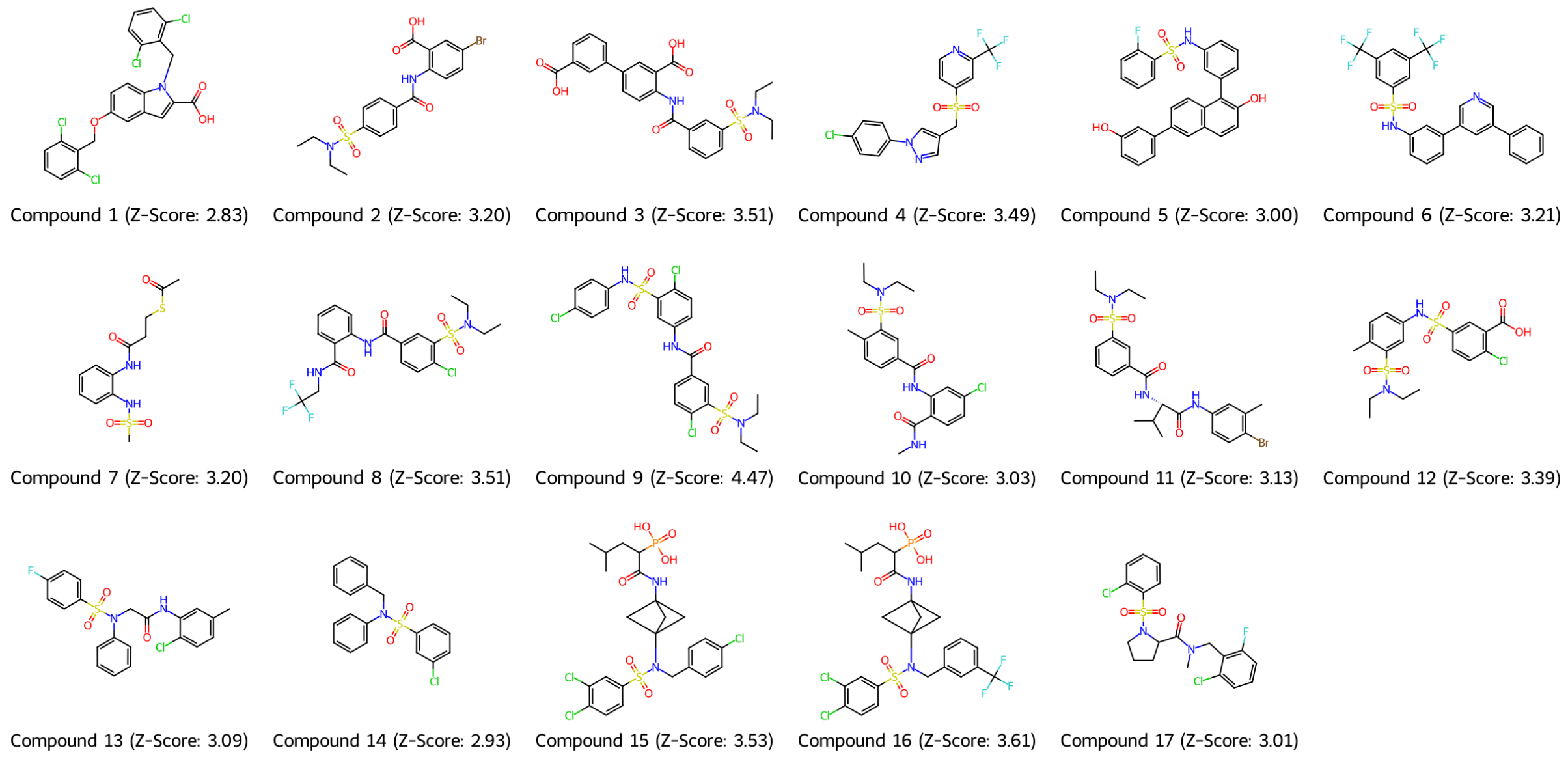

Supplementary Figure 8. Two-dimensional structures of the seventeen MolDockLab-selected compounds advanced to in vitro testing. Chemical structures of the seventeen compounds selected by MolDockLab for the energy-coupling factor transporter and advanced to in vitro validation. Each structure is labelled with its compound identifier and the consensus Z-score assigned by the MolDockLab ranking pipeline.
